# Interfacially engineered metal oxide nanocomposites for enhanced photocatalytic degradation of pollutants and energy applications

**DOI:** 10.1039/d4ra08780a

**Published:** 2025-05-12

**Authors:** Mahmoud A. Ahmed, Safwat A. Mahmoud, Ashraf A. Mohamed

**Affiliations:** a Chemistry Department, Faculty of Science, Ain Shams University Cairo-11566 Egypt mahmoudmahmoud_p@sci.asu.edu.eg; b Center for Scientific Research and Entrepreneurship, Northern Border University Arar 73213 Saudi Arabia

## Abstract

Escalating global energy demands and environmental pollution necessitate innovative solutions for sustainable development. Conventional methods often prove inadequate, driving research towards advanced materials and technologies. This review critically analyzes existing industrial wastewater treatment approaches, highlighting their merits and limitations, before focusing on the recent advancements in metal oxide-based nanocomposite photocatalysis for both pollutant degradation and energy generation. Moreover, the structural, electronic, and optical properties of metal oxides (MOx) are elucidated. The review discusses various MOx synthesis routes and their nanocomposites and elucidates the underlying photocatalytic mechanisms, emphasizing the influence of operational parameters on photocatalytic efficiency. Moreover, it explores how MOx can be utilized for photocatalytic energy generation, in addition to their role in pollutant degradation. Furthermore, it delves into the synergistic effects achieved by combining MOx with complementary nanomaterials (carbon-based structures, polymers, non-metals, semiconductors, and metal sulfides) to create hybrid nanocomposites with enhanced photocatalytic activity for both applications. A cost analysis and SWOT analysis are presented to assess the economic and technological feasibility of this trend. This comprehensive overview provides valuable insights for developing efficient, sustainable, and scalable wastewater treatment solutions using MOx-based nanocomposites, ultimately contributing to improved environmental remediation and water resource management while simultaneously exploring opportunities for energy production.

## Abstract 2

Escalating global energy demands and environmental pollution necessitate innovative solutions for sustainable development.

## Graphical abstract

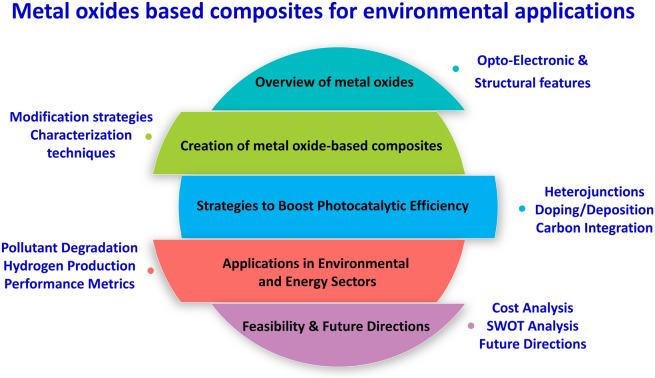

## 1. Introduction

The confluence of escalating global energy demands and the pervasive ramifications of environmental pollution presents a formidable challenge to sustainable development.^1–4^ The continued reliance on fossil fuels poses a substantial threat to energy security and exacerbates environmental degradation.^5^ The combustion of fossil fuels releases copious amounts of greenhouse gases, primarily carbon dioxide, contributing to global warming and its associated implications, including extreme weather events, rising sea levels, and disruptions to delicate ecological balances.^6,7^ Moreover, the extraction, processing, and utilization of fossil fuels generate a plethora of pollutants, including sulfur oxides (SOx), nitrogen oxides (NOx), particulate matter (PM), and volatile organic compounds (VOCs), which contribute to air pollution, acid rain, and respiratory complications.^8,9^ Simultaneously, the discharge of a diverse array of pollutants from industrial activities, agricultural practices, and urbanization poses a grave threat to environmental integrity and human health. Industrial effluents often contain heavy metals, pharmaceuticals, dyes, and persistent organic pollutants (POPs), contaminating water bodies and disrupting aquatic ecosystems.^10–15^ Agricultural runoff laden with pesticides, fertilizers, and herbicides contributes to eutrophication, soil degradation, and water pollution. This precarious scenario necessitates a paradigm shift towards clean and renewable energy sources coupled with effective pollution mitigation strategies.

Various effluent treatment methods (*e.g.*, biological processes, coagulation–flocculation, sedimentation, disinfection, ion exchange, membrane filtration) are often insufficient for complete removal of recalcitrant pollutants.^16–19^ These methods suffer from limitations such as fouling, high operating pressures; concentrate stream generation, and inefficient removal of low concentrations of emerging pollutants. Consequently, advanced oxidation processes (AOPs) have emerged as a promising alternative.^20^ AOPs generate highly reactive hydroxyl radicals (˙OH) that non-selectively mineralize a wide range of organic pollutants to CO_2_, H_2_O, and inorganic salts. Various AOPs exist, including photocatalysis, ozonation, sonochemical, Fenton, photoFenton, sonophoto-Fenton, and electrochemical oxidation processes.^21,22^ Among these, photocatalysis is particularly attractive due to its potential to mitigate both energy scarcity and environmental pollution by utilizing solar or artificial light to activate a semiconductor material, generating ˙OH and other reactive oxygen species (ROS) for targeted catalytic reactions.

**Fig. 1:**
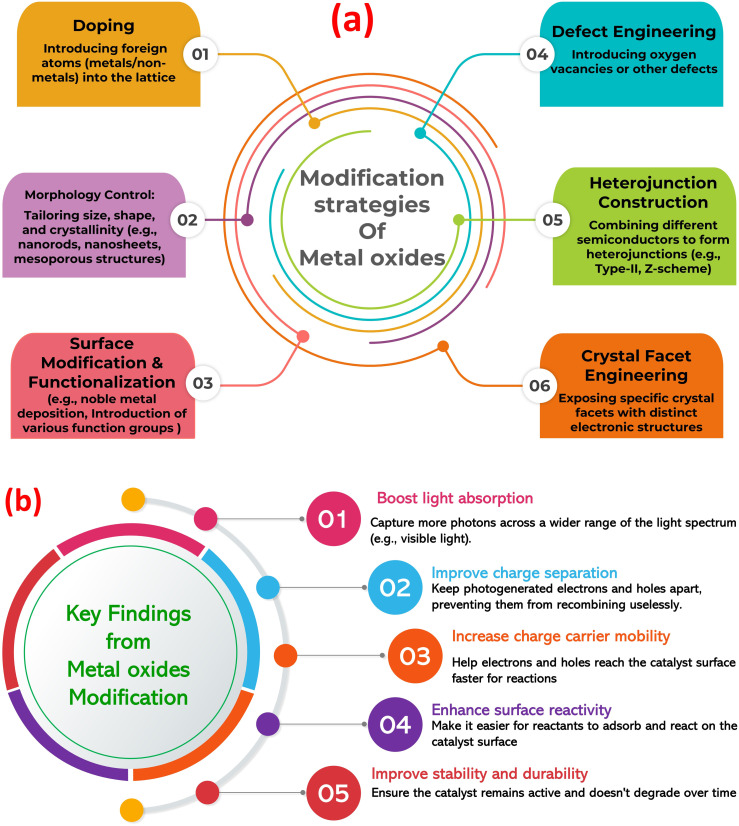
(a) Modification approaches of metal oxides to enhance its photocatalytic role, (b) the key findings from the modification of metal oxide photocatalysts.

Metal oxides are a diverse class of materials with a wide range of optical, structural, and electronic properties, making them crucial for various technologies.^23–26^ Their diverse functionalities arise from the interplay between metal and oxygen ions, influenced by the metal's oxidation state, coordination geometry, and crystal structure. Metal oxides exhibit diverse structures, from simple rock salt (*e.g.*, MgO) to complex perovskite (*e.g.*, SrTiO_3_) and layered structures (*e.g.*, MoO_3_).^27–29^ This structural diversity significantly impacts their electronic band structure and optical properties. Closely packed structures often lead to wide band gaps and transparent/insulating behavior, while open structures with transition metals can exhibit smaller band gaps, resulting in semiconducting or metallic conductivity. Defects, like oxygen vacancies, further modulate the electronic structure, influencing optical absorption and conductivity.^30,31^ The band gap, the energy difference between valence and conduction bands, determines the minimum photon energy for electron excitation (absorption edge). Transition metal oxides often absorb visible light and exhibit characteristic colors due to partially filled d-orbitals and phenomena like d–d transitions, charge transfer transitions, and plasmon resonances.^32–34^ This unique tunability makes metal oxides (MOx) a compelling platform for photocatalysis, using light to drive chemical transformations. MOx are attractive due to their favorable band structures, cost-effectiveness, abundance, and chemical stability. Photocatalysis relies on MOx absorbing photons to generate electron–hole pairs that drive redox reactions. However, limitations like nanoparticles agglomeration, rapid electron–hole recombination, limited visible light absorption, and low charge carrier mobility hinder pristine MOx performance. To overcome these challenges, strategies such as doping, creating heterojunctions, surface functionalization, and morphology control are employed, with the construction of MOx-based composites gaining significant attention (Fig. 1a).^35–40^ Further, Fig. 1b highlights the key findings from the modification of MOx photocatalysts. Combining MOx with other materials (semiconductors, carbonaceous nanomaterials, noble metals, or polymers) creates synergistic effects that enhance photocatalytic activity. These composites improve charge separation, broaden light absorption (*via* sensitizers or plasmon resonances), increase surface area and dispersibility, and enhance MO stability. Therefore, designing MO-based composites is crucial for realizing the full potential of metal oxide photocatalysis in applications from environmental remediation to energy conversion.

**Fig. 2:**
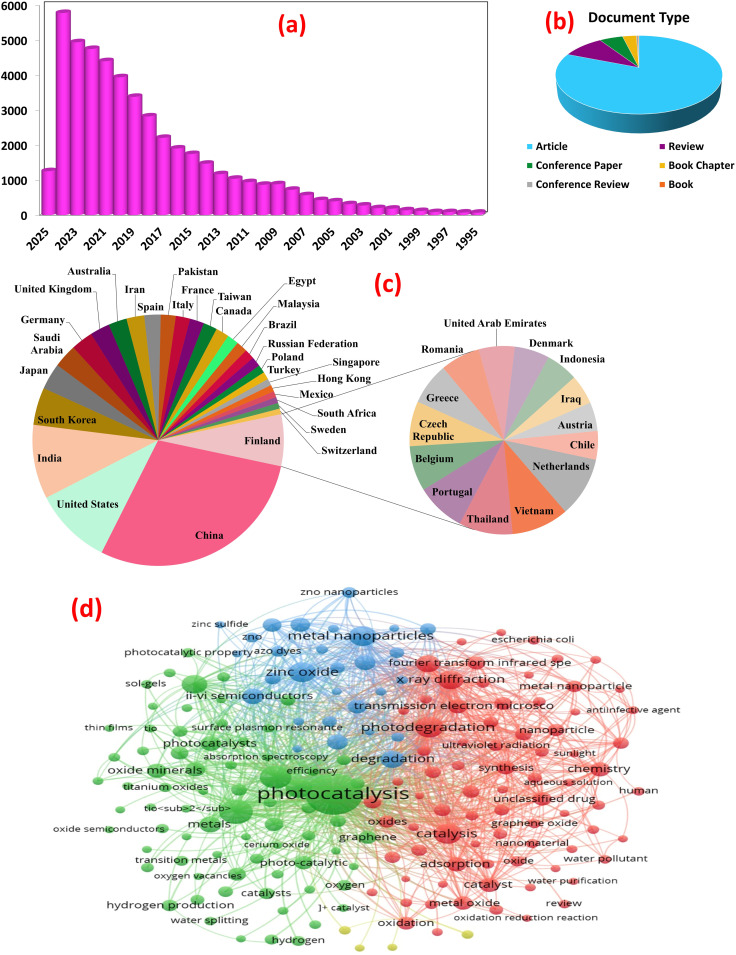
Trends in publications over the last four decades that analyze SCOUPS data; keywords are “metal oxide”, “pollutants”, “photocatalysis”, “water splitting”, “energy conversion”, “hydrogen production”, and “wastewater treatment”. The figure includes: (a) yearly publication trends, (b) document type, (c) contributions from various countries to the field, and (d) bibliometric network analysis map of keywords.

Analysis using Scopus data (accessed February 27, 2025) mapped the evolving landscape of metal oxide nanocomposite photocatalysis for wastewater treatment. Employing keywords: “metal oxide”, “pollutants”, “photocatalysis”, “water splitting”, “energy conversion”, “hydrogen production”, “wastewater treatment”, *etc.*, 48 018 documents were retrieved, encompassing 38 725 research articles, 4642 reviews, and other publication types, reflecting the exponential growth in publications, particularly over the past decade, underscoring the burgeoning interest in this field, as shown in Fig. 2a and b. A bibliometric analysis of keywords from photocatalysis articles in PubMed reveals core research areas and emerging trends, Fig. 2d. Network visualization clustered the keywords based on co-occurrence frequency. The first cluster centers on fundamental photocatalytic mechanisms. It focuses on photocatalytic degradation of organic pollutants (*e.g.*, dyes, pharmaceuticals) using MOx like ZnO and TiO_2_ under visible light. The research prioritizes enhancing activity through nano-structural optimization (*e.g.*, particle size, morphology) and addressing inherent limitations like rapid electron–hole recombination. Recent advancements integrate MOx with other materials (*e.g.*, graphene) to improve visible light absorption and charge transfer efficiency. Heterojunctions with transition metal oxides (Fe_2_O_3_, CuO) or noble metals (Ag nanoparticles) further narrow bandgaps, enabling dual-functionality for organic pollutant degradation and heavy metal removal (*e.g.*, Cr, Cd) *via* adsorption-photocatalytic mechanisms. The second cluster explores MOx composites (*e.g.*, MnO_2_–Fe_2_O_3_ hybrids, graphene-supported TiO_2_) as electro- and photo-catalysts for hydrogen production. Enhanced charge separation and catalytic activity under solar irradiation are achieved through conductive matrices (*e.g.*, carbon nanorods), optimizing electron transport for efficient oxygen/hydrogen evolution reactions. Interdisciplinary approaches combining photocatalysis with electrocatalysis aim to scale solar-driven hydrogen production, aligning with global clean energy goals. Material engineering, *via* sol–gel methods, pyrolysis, and green synthesis, enables precise control over nanocomposite properties (*e.g.*, crystallinity, porosity) to maximize active sites. Characterization tools guide structure–activity relationships, while eco-friendly synthesis and recycling address sustainability. Challenges include scalability, long-term stability, and mitigating secondary pollution from metal leaching. Future research may leverage bio-inspired designs, machine learning, and pilot-scale trials to bridge lab innovations with real-world applications. This analysis underscores the transformative role of metal oxide composites in advancing sustainable water treatment and renewable energy systems.

Therefore, the present review provides a critical appraisal of current and emerging technologies for wastewater remediation, focusing on the burgeoning field of MOx-based nanocomposite photocatalysis. Beyond simply summarizing recent advancements, this work dissects the fundamental photocatalytic mechanisms governing enhanced performance in these materials, elucidating the intricate interplay between material design, synthetic strategies, and operational parameters. It explores the synergistic impacts arising from the integration of MOx with diverse nanomaterials, highlighting the resulting improvements in photocatalytic activity for both pollutant degradation and energy generation. A key objective is to address the critical research gap related to the long-term stability and recyclability of MOx nanocomposite photocatalysts in wastewater treatment, evaluating current strategies and proposing innovative approaches for enhancing their practical applicability. Furthermore, it explores emerging strategies to overcome the associated limitations and propose future research directions to unlock the full potential of MOx nanocomposites for sustainable wastewater treatment and resource recovery, paving the way for a circular economy approach to water management.

## 2. Optical features and electronic structure of some metal oxides

MOx display intriguing optical features and possesses a distinctive electronic structure, rendering it a subject of great interest in numerous scientific disciplines.^41^ For instance, NiO is optically renowned for its characteristic deep green color, which arises from its electronic transitions within the visible range.^42,43^ The absorption spectrum of NiO exhibits pronounced absorption in the ultraviolet (UV) region, extending into the visible range.^44–46^ This absorption is attributed to charge transfer transitions between the valence band originating mainly from oxygen 2p orbitals to the conduction band originating mainly from nickel 3d orbitals. The bandgap energy of NiO typically resides around 3.4–4.0 eV, classifying it as a wide-bandgap material suitable for applications in the UV region.^47–49^ The electronic structure of NiO is intricately linked to its cubic crystal structure, which typically adopts a rock salt-like configuration (NaCl structure). In this arrangement, each nickel ion is encompassed by six oxygen ions in an octahedral coordination. The electronic configuration of nickel in NiO is [_18_Ar]3d^8^, with two unpaired electrons occupying the 3d orbitals.^50^ NiO is also recognized as a p-type semiconductor.^51^ This means that it exhibits a predominance of positive charge carriers, known as “holes”, in its electronic structure.^52^ The p-type behavior of NiO arises from the presence of oxygen vacancies and nickel interstitials, which create acceptor states within the bandgap. The presence of oxygen vacancies or other defects can perturb the band structure, thereby influencing the optical and electronic properties of NiO. Many studies have examined the effect of oxygen vacancies on the reactivity of NiO in applications like catalysis, photocatalysis, sensing, and electronic devices.^53–56^ These studies used computational and experimental approaches to investigate the impact of oxygen vacancies on the performance of NiO. The primary limitation of NiO semiconductors in photocatalytic processes is its quick charge carriers recombination and wide band gap, which restrict its performance in the visible region. This poses a challenge for the degradation of complex organic molecules, as it necessitates efficient photon generation and charge carrier separation to initiate effective radical-driven redox reactions.

Titanium dioxide (TiO_2_) stands as a prominent metal oxide, extensively studied and utilized due to its diverse and tunable properties.^57^ TiO_2_ exists primarily in three crystalline polymorphs: anatase, rutile, and brookite.^58^ Anatase, characterized by a tetragonal structure with edge-sharing TiO_6_ octahedra, is generally considered as the most photocatalytically active phase due to its higher electron mobility and longer charge carrier lifetime compared to rutile.^59,60^ Rutile, also tetragonal but with a more compact structure featuring both edge- and corner-sharing octahedra, exhibits a higher refractive index and greater UV absorption, making it suitable for pigments and UV-blocking applications.^61–63^ Brookite, with its orthorhombic structure, has shown promising photocatalytic activity in specific reactions but is often challenging to synthesize in pure form. The structural differences between these polymorphs directly influence their electronic band structures and consequently their optical properties. TiO_2_'s optical properties are dominated by its semiconducting nature. The band gap energies of anatase (∼3.2 eV) and rutile (∼3.0 eV) correspond to absorption in the near-UV region, resulting in their white appearance and excellent UV-blocking capabilities.^64^ The electronic transitions responsible for this absorption involve the excitation of electrons from the O 2p valence band to the Ti 3d conduction band. Defects within the TiO_2_ lattice, such as oxygen vacancies and Ti^3+^ interstitials, can introduce localized states within the band gap, influencing the optical absorption and photocatalytic activity.^64,65^ For example, oxygen vacancies can create shallow donor levels below the conduction band, enhancing visible light absorption and potentially increasing photocatalytic efficiency.^66^ Furthermore, the refractive index of TiO_2_, particularly in the rutile phase, is relatively high (∼2.4–2.9), making it a valuable material for optical coatings and anti-reflection layers.^67^

Zinc oxide (ZnO), a versatile II–VI semiconductor, presents a compelling platform for photocatalytic applications.^68^ Crystalline ZnO predominantly adopts the wurtzite structure, characterized by a hexagonal unit cell with tetrahedrally coordinated Zn^2+^ and O^2−^ ions.^69^ This non-centrosymmetric arrangement gives rise to intrinsic piezoelectric and pyroelectric properties, potentially influencing charge separation and photocatalytic activity. ZnO's direct band gap of ∼3.37 eV at room temperature dictates its optical absorption in the near-UV region, rendering it transparent in the visible spectrum.^70^ This character, while advantageous for certain applications like UV filters, limits its utilization of the full solar spectrum for photocatalysis. However, the high exciton binding energy (∼60 meV) in ZnO results in robust excitonic absorption features even at room temperature, suggesting efficient exciton formation and potential for enhanced photocatalytic activity through exciton-mediated processes.^71,72^ Furthermore, defects inherent to ZnO, such as oxygen vacancies and zinc interstitials, play a pivotal role in defining its electronic properties, often contributing to n-type conductivity.^73,74^ These defects can also introduce localized states within the band gap, influencing charge carrier dynamics and potentially impacting photocatalytic performance. The electronic structure of ZnO, characterized by a filled O 2p valence band and an empty Zn 4s conduction band, governs its photocatalytic behavior. Upon UV irradiation, electrons are excited across the band gap, creating electron–hole pairs that can participate in redox reactions at the ZnO surface. The efficiency of this process, however, is often limited by the rapid recombination of these charge carriers. Furthermore, the relatively high reduction potential of photogenerated electrons in ZnO restricts its ability to reduce certain species, limiting its applicability in some photocatalytic reactions. Despite these challenges, ZnO's high electron mobility, large surface area-to-volume ratio in nanostructured forms, and relatively low toxicity make it an attractive candidate for photocatalysis. Moreover, its inherent photostability compared to some other metal oxides further strengthens its potential for sustained photocatalytic activity. Understanding the interplay between ZnO's intrinsic properties, including its crystal structure, electronic band structure, defect chemistry, and optical absorption characteristics, is crucial for developing strategies to enhance its photocatalytic performance.

CuO crystallizes in a monoclinic structure, characterized by Cu^2+^ ions in a distorted square planar coordination with oxygen.^75^ This structural arrangement contributes to its distinct electronic properties and influences its interaction with light. CuO is a p-type semiconductor with a relatively narrow band gap, typically ranging from 1.2 to 1.9 eV, depending on the synthesis method and particle size.^76–78^ Furthermore, CuO exhibits strong absorption in the visible and near-infrared regions due to its narrow band gap and d–d electronic transitions within the Cu^2+^ ions. The absorption characteristics can be further influenced by factors such as particle size, morphology, and crystal defects. Nanostructured CuO, for instance, can exhibit enhanced optical absorption due to increased surface area and quantum confinement effects. The optical properties of CuO are also relevant for applications beyond photocatalysis, including solar cells, gas sensors, and electrochromic devices. Moreover, CuO is characterized by its p-type semiconductivity, arising from the presence of copper vacancies, which act as acceptor levels. The relatively high hole mobility in CuO facilitates charge transport, but the rapid recombination of photogenerated electron–hole pairs limits its photocatalytic efficiency.

WO_3_ exhibits polymorphism, adopting different crystal structures (monoclinic, orthorhombic, tetragonal, and cubic) depending on temperature and synthesis conditions.^79,80^ The monoclinic phase is stable at room temperature, and is most commonly investigated for photocatalysis.^81,82^ Crucially, the WO_3_ lattice readily accommodates oxygen vacancies, creating WO_3−*x*_, where the degree of oxygen deficiency (*x*) significantly influences its electronic structure and, consequently, its optical and electronic properties. These oxygen vacancies introduce defect states within the band gap, impacting charge carrier behavior and overall photocatalytic activity.^83^ Moreover, WO_3_ is an n-type semiconductor with a band gap typically ranging from 2.6 to 3.0 eV for the stoichiometric composition, corresponding to absorption in the visible to near-UV region.^84,85^ The presence of oxygen vacancies, however, plays a pivotal role in modulating the electronic band structure. These vacancies introduce localized states within the band gap, effectively narrowing the band gap energy and extending the absorption spectrum further into the visible light region. This shift towards visible light absorption is highly desirable for solar-driven photocatalysis, as it enables more efficient utilization of the solar spectrum. Furthermore, the presence of these defect states can influence charge carrier dynamics, affecting both charge separation and recombination rates, which are critical parameters governing photocatalytic efficiency. Further, the most striking feature of WO_3_ is its electrochromic behavior, stemming from the reversible insertion and extraction of ions, often accompanied by changes in oxygen vacancy concentration. This process modulates the optical absorption properties, leading to a dramatic and reversible color change, ranging from transparent or pale yellow in the oxidized state to deep blue or black in the reduced state. This dynamic optical tunability, while exploited in electrochromic devices, also has implications for photocatalysis. The precise control over oxygen vacancies, and therefore the optical absorption, allows for tailoring the light absorption properties to match the desired spectral range for specific photocatalytic reactions. Moreover, the high refractive index of WO_3_ can be advantageous in certain photocatalytic configurations, enhancing light trapping within the material and potentially increasing the interaction of light with the photoactive sites.

Cerium dioxide (CeO_2_) stands as a compelling metal oxide with unique redox properties stemming from the facile switching between Ce^4+^ and Ce^3+^ oxidation states.^86,87^ CeO_2_ adopts a fluorite crystal structure, characterized by a face-centered cubic arrangement of Ce^4+^ cations and O^2−^ anions.^88,89^ This structure facilitates the formation of oxygen vacancies, which are crucial for the material's redox activity and catalytic performance. The presence of oxygen vacancies and the associated Ce^3+^ ions introduce localized states within the band gap, influencing the optical absorption and electronic conductivity. Furthermore, the ability of CeO_2_ to readily store and release oxygen makes it an effective oxygen buffer, a property that is exploited in various catalytic applications, including three-way catalysts for automotive exhaust gas treatment. Typically, CeO_2_ is pale yellow to off-white in color due to its absorption edge in the near-UV region.^90,91^ The band gap of CeO_2_ is generally reported to be around 3.2 eV, although the precise value can vary depending on the synthesis method and the presence of defects.^91^ The absorption edge arises from charge transfer transitions between the O 2p valence band and the Ce 4f conduction band. The presence of oxygen vacancies and Ce^3+^ ions introduces defect states within the band gap, leading to increased absorption in the visible region. This enhanced visible light absorption can be advantageous for photocatalytic applications, as it allows for utilization of a broader portion of the solar spectrum. Furthermore, the refractive index of CeO_2_ is relatively high, making it suitable for optical coatings. Electronically, CeO_2_ exhibits n-type semiconducting behavior, with the conductivity largely influenced by the concentration of oxygen vacancies and Ce^3+^ ions. The presence of these defects introduces donor levels within the band gap, increasing the electron carrier concentration. The electronic conductivity of CeO_2_ can be further tuned by doping with other elements or by controlling the oxygen vacancy concentration through annealing under different atmospheres. The facile switching between the Ce^4+^/Ce^3+^ oxidation states allows CeO_2_ to participate in redox reactions, acting as an oxygen buffer and promoting the activation of reactants. This redox activity is particularly important in photocatalysis, where CeO_2_ can promote charge separation and enhance the efficiency of redox reactions at the surface.

Co_3_O_4_ crystallizes in the normal spinel structure, where Co^2+^ ions occupy tetrahedral sites and Co^3+^ ions occupy octahedral sites within a cubic close-packed oxygen lattice.^92,93^ This specific cation distribution and the interplay between the two cobalt oxidation states significantly influence the electronic, magnetic, and catalytic properties of Co_3_O_4_. Its electronic structure is characterized by a complex interplay of electron correlations and spin–orbit coupling, leading to interesting phenomena such as antiferromagnetic ordering at low temperatures. Co_3_O_4_ exhibits two prominent absorption bands in the visible region, at around 400 and 700 nm, respectively.^93,94^ These absorption features arise from ligand-to-metal charge transfer transitions involving O^2−^ and Co^2+^/Co^3+^ ions. The first absorption band is attributed to O^2−^ → Co^3+^ transitions, while the second band is associated with O^2−^ → Co^2+^ transitions. The precise position and intensity of these absorption bands can be influenced by factors such as particle size, morphology, and the presence of defects. The optical properties of Co_3_O_4_ make it a potential candidate for applications in solar energy conversion and photocatalysis.^95^ Furthermore, the refractive index and extinction coefficient of Co_3_O_4_, crucial parameters for optical applications, can be tuned by controlling the synthesis conditions and morphology. Moreover, Co_3_O_4_ exhibits p-type semiconducting behavior with a band gap typically ranging from 1.4 to 2.2 eV, depending on the synthesis method and morphology.^96,97^ The relatively small band gap allows for absorption of a significant portion of the visible light spectrum, making it suitable for photocatalytic applications. The electrical conductivity of Co_3_O_4_ is influenced by the concentration of oxygen vacancies and other defects, which can act as charge carriers. The unique electronic structure of Co_3_O_4_, with the coexistence of Co^2+^ and Co^3+^ ions, facilitates redox reactions, making it a promising catalyst for various applications, including CO oxidation, oxygen evolution reaction (OER), and the degradation of organic pollutants. Furthermore, Co_3_O_4_ has shown potential for application in energy storage devices, such as supercapacitors and lithium-ion batteries, due to its good electrochemical performance and relatively low cost.

**Fig. 3:**
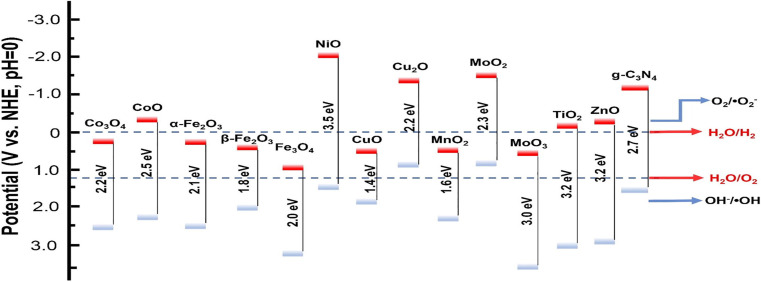
Energy levels of the CB and VB of some metal oxides, compared to the redox potentials of water splitting and selected free radicals (*versus* NHE) at pH = 0, reprinted with the permission of ref. 98, copyright 2025, Elsevier.

Fig. 3 illustrates the bandgap energies along with the CB and valence VB edge positions of some metal oxides.^98^ The data highlight variations in electronic structure, which are critical for understanding their photocatalytic and optoelectronic properties. Precise alignment of CB and VB levels relative to redox potentials (*e.g.*, water oxidation/reduction levels) is emphasized, as this directly influences charge transfer efficiency in applications such as solar energy conversion and pollutant degradation.

## 3. Interfacial engineering of metal oxides and their composite photocatalysts

Interfacial engineering plays a pivotal role in enhancing the performance of metal oxides and their composite photocatalysts. The interface between the composite components is crucial for charges creation and separation, and surface reactions. We can significantly improve photocatalytic efficiency for various applications, including environmental remediation, water splitting, and organic synthesis, by carefully modifying the interface structure, composition, and properties. This can be achieved *via*, for example well-tailored synthesis methods, heterojunction formation, doping with metallic and nonmetallic species, enhancing the surface area and surface functional groups, creating surface defects and oxygen vacancies and their densities, improving light harvesting, especially in the visible region, lowering bandgap energies, and improving photocatalyst stability and reusability.

### 3.1 Fabrication methods for metal oxide-based composites

**Fig. 4:**
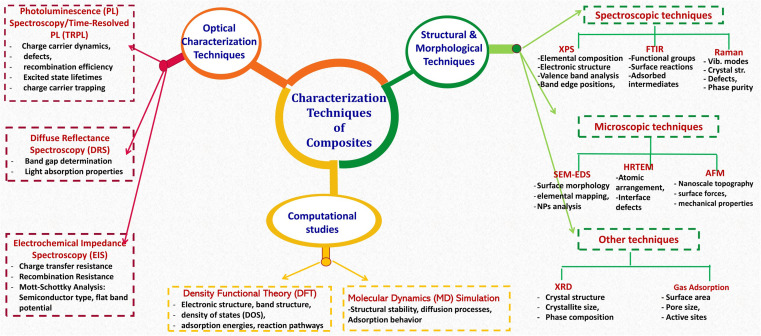
Summary of characterization techniques of MOx-based materials.

The synthesis of metal oxides-based composites for environmental applications leverages a range of preparative techniques, broadly categorized as *in situ* or *ex situ* crystallization strategies. *In situ* methods entail the simultaneous formation of both the metal oxides and the composite matrix, promoting a homogeneous distribution of metal oxides and strong interfacial interactions with the supporting material. This approach often allows for finer control over crystallite size and morphology, potentially leading to enhanced performance. Conversely, *ex situ* methods involve the incorporation of pre-synthesized metal oxide nanoparticles into a separate matrix material. This offers flexibility in tailoring the properties of both components independently before composite formation, although achieving uniform dispersion and preventing particle agglomeration can be challenging. Several well-established techniques are employed within both strategies, *e.g.*, sol–gel, hydrothermal and solvothermal methods, co-precipitation microwave-assisted, ultrasound-assisted methods. After a successful synthesis of MOx-based composites, multifaceted analysis and detailed characterization techniques are important for understanding the properties of compounds and the structure–property relationships. Fig. 4 summarizes common characterization techniques used to analyze the structural, optical, and electronic properties of fabricated composite photocatalysts.

**Fig. 5:**
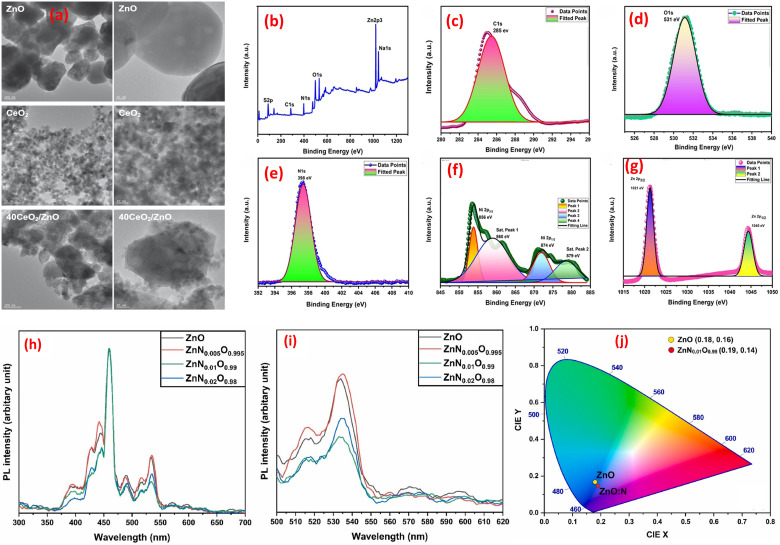
(a) HRTEM images of representative ZnO, Ce_2_O, and Ce_2_O/ZnO composite, reprinted with the permission of ref. 103 copyright 2025, Elsevier; (b–g) XPS analysis of the NiO/ZnO/g-C_3_N_4_ nanocomposite material: (b) full survey spectrum, (c) C 1s, (d) N 1s, (e) Ni 2p, (f) Zn 2p, and (g) O 1s core-level spectra, reprinted with the permission of ref. 104, copyright 2025, Elsevier; (h) PL spectra (300–700 nm), (i) detailed emission profiles (500–620 nm) for varying nitrogen concentrations of ZnO and nitrogen-doped ZnO (ZnN_*x*_O_1−*x*_ NPs), and (j) CIE chromaticity coordinates comparing color emission characteristics of ZnO and ZnN_0.01_O_0.99_ NPs, reprinted with the permission of ref. 105, copyright 2025, Elsevier.

Co-precipitation is a foundational wet chemical method for synthesizing metal oxide-based composites, enabling precise control over physicochemical properties by tuning reaction parameters.^99^ This process involves simultaneous precipitation of multiple metal cations from a solution using agents like hydroxide (OH^−^), carbonate (CO_3_^2−^), or oxalate (C_2_O_4_^2−^) ions.^100,101^ Precise pH control ensures homogeneous cation co-precipitation by balancing hydrolysis equilibria, while the choice of agent dictates precursor crystallinity; *e.g.*, hydroxides yield amorphous phases, while carbonates form ordered frameworks. Precipitation kinetics, governed by concentration, mixing, and temperature, modulate particle morphology: slow nucleation favors monodisperse crystallites (Ostwald ripening), while rapid quenching produces metastable nanoparticles. Calcination thermally transforms precursors; temperature regulates crystallinity and sintering, while atmosphere (*e.g.*, O_2_, H_2_/N_2_) tunes oxidation states and oxygen vacancies, enhancing functionality for catalysis, sensing, or energy storage *via* defect-engineered charge transport and surface reactivity. For example, a CeO_2_/ZnO nanocomposite, synthesized *via* co-precipitation, exhibited roughly double the activity of ZnO alone and ten times the activity of pure CeO_2_.^102^ The synthesized catalyst morphology was analyzed *via* HRTEM, as shown in Fig. 5a, showing that spherical-shaped and crystalline ZnO nanoparticles (5–200 nm) were decorated with smaller CeO_2_ NPs (8–17 nm). The 40CeO_2_/ZnO hybrid comprised aggregated ZnO and CeO_2_ phases forming a porous structure. Separately, a ternary NiO/ZnO/g-C_3_N_4_ composite was developed and tested for its azo dye degradation capabilities.^103^ The two-dimensional structure of g-C_3_N_4_ provided ample nucleation sites, facilitating the growth of nickel oxide and zinc oxide NPs. The resulting NZC nanocomposite displayed a clustered, curled morphology.^103^ XPS analysis, shown in Fig. 5b–g, confirmed that the NiO/ZnO/g-C_3_N_4_ composite contains C (C–N bonds in g-C_3_N_4_), N (C–N–C/N–(C)_3_ groups), Ni^2+^ (NiO), Zn^2+^ (ZnO), and O (Ni–O/Zn–O bonds), with oxygen-deficient defects, validating its chemical structure. Further, iron-doped ZnO was fabricated *via* chemical co-precipitation.^104^ XRD characterization revealed a single-phase crystalline structure for both the undoped and iron-doped ZnO NPs. The SEM imaging showed agglomerates of NPs with varying sizes. A minimization in the band gap energy was noticed with increasing iron content, attributed to modifications in the lattice parameters.^104^ Further, the synthesis of nitrogen-doped ZnO was reported, noting the development of a nanorod morphology upon nitrogen incorporation.^106^ Photoluminescence (PL) analysis revealed nitrogen-doped ZnO nanoparticles exhibit band gap narrowing, reduced electron–hole recombination at 1% doping (*via* non-radiative centers), and enhanced UV-blue emission (CIE chromaticity shift), making them suitable for UV-light devices, Fig. 5h and i. Additionally, the growth of CuO/CdO nanosheets using a co-precipitation route was described.^105^ The introduction of the non-ionic surfactant Triton X-100 led to the formation of hexagonal, nanoporous grains. While pristine CuO and CdO exhibited monoclinic and cubic crystal structures, respectively, XRD analysis of the CuO/CdO composite revealed a mixed-phase composition.^105^

**Fig. 6:**
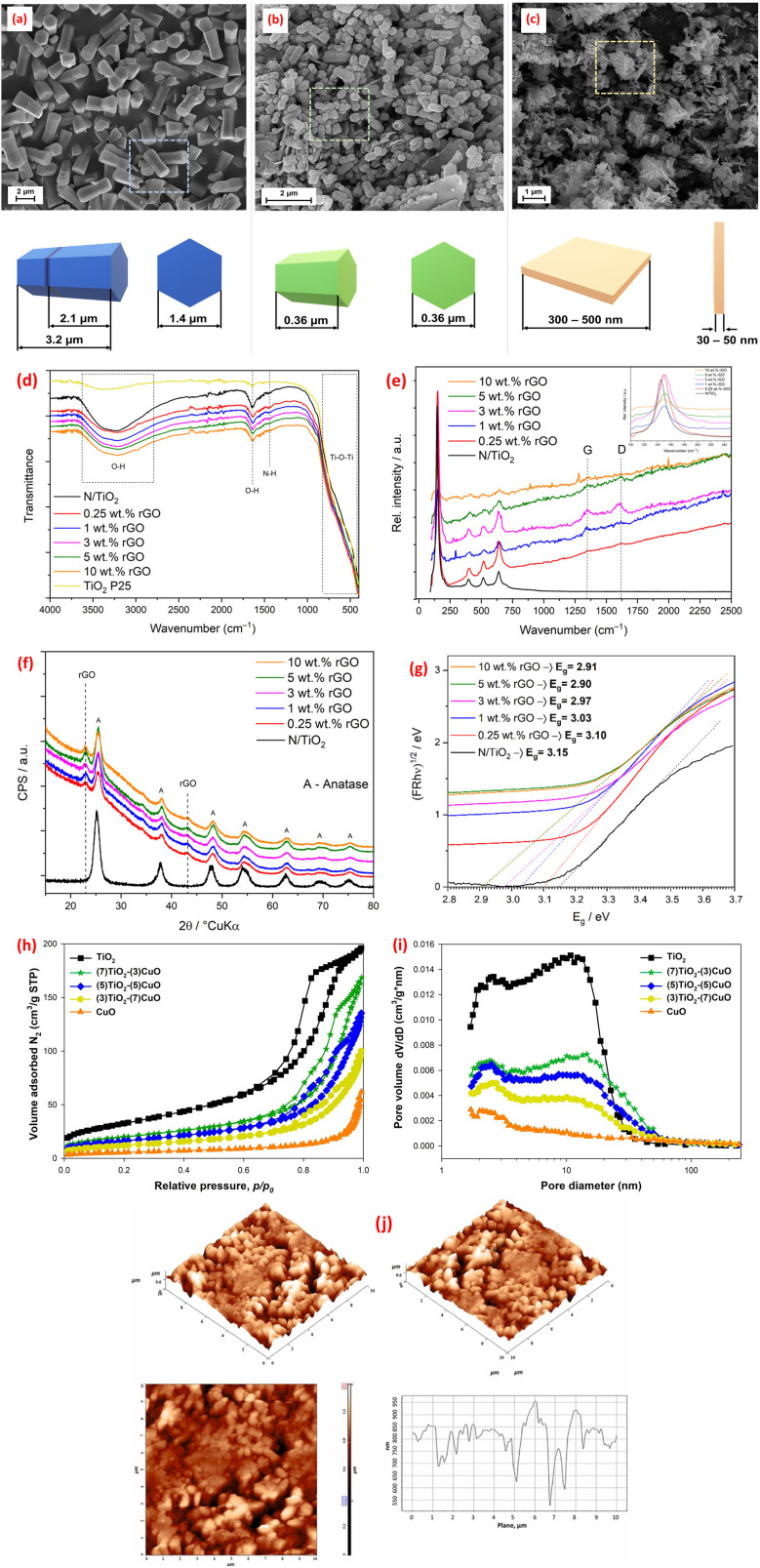
SEM images for ZnO samples obtained at pH: (a) 8; (b) 10; and (c) 12, reprinted with the permission of ref. 108, copyright 2025, Elsevier; (d) FTIR and (e) Raman spectra, (f) XRD patterns, and (g) reprinted with the permission of ref. 109, copyright 2025, Elsevier; Tauc plots for bandgap analysis of N/TiO_2_ and N/TiO_2_/rGO photocatalysts with varying rGO loadings (0.25–10 wt%), (h and i) N_2_ adsorption/desorption isotherms and pore diameter distributions of fabricated TiO_2_, CuO, and TiO_2_/CuO composites, reprinted with the permission of ref. 110, copyright 2025, Elsevier; (j) AFM images and AFM roughness profile of CeO_2_@ZnO CNS, reprinted with the permission of ref. 111, copyright 2025, Elsevier.

Microwave-assisted synthesis has emerged as a powerful technique leveraging electromagnetic radiation in the frequency range of 300 MHz–300 GHz to drive chemical reactions.^46^ This method offers distinct advantages, including cost-effectiveness, time efficiency, energy savings, and the ability to produce controlled-size products.^46^ Unlike conventional heating methods, microwave irradiation facilitates rapid and homogeneous heating of materials, leading to optimal nucleation conditions, short crystallization times, and enhanced control over macroscopic morphology and size distribution during fabrication.^46^ This precise control over the synthetic environment allows for the tailoring of metal oxides composite properties, making microwave-assisted methods particularly attractive for optimizing performance in environmental applications. Thus, a CoFe_2_O_4_@TiO_2_@rGO nanocomposite (CoTG) was synthesized using a combination of microwave and sol–gel methods. TEM analysis confirmed the successful impregnation of spherical TiO_2_ and CoFe_2_O_4_ nanoparticles onto rGO sheets.^107^ DRS spectra revealed that the CoTG nanocomposite exhibited an effective bandgap for visible light activity.^107^ Furthermore, solution pH significantly influenced the crystallinity and structure of ZnO synthesized using microwave irradiation, as demonstrated by the SEM micrographs in Fig. 6a–c.^108^ Similarly, the photocatalyst morphology exhibited a strong pH-dependency.^112^ Furthermore, a cost-effective microwave-assisted method as employed to create N-doped TiO_2_/rGO hybrid composites (N/TiO_2_/rGO) with varying rGO content.^109^ Anatase was the only crystalline phase detected in the synthesized materials. While rGO loading did not affect morphology, it improved photocatalytic activity, especially at lower concentrations.^109^ The FTIR, Raman, XRD, and DRS spectra, shown in Fig. 6d–g, confirmed the anatase TiO_2_ structure in N/TiO_2_/rGO composites, with Raman and XRD indicating rGO integration (*via* D/G bands and disorder peaks) and FTIR showing reduced O–H intensity due to rGO's hydrophobicity. Tauc plot revealed rGO reduces the bandgap (optimal at ∼5 wt%), enhancing charge separation and narrowing the bandgap for improved photocatalytic activity (Fig. 6g). Additionally, the successful microwave synthesis of TiO_2_–CuO materials with well-defined crystalline structures was reported.^110^ Nitrogen adsorption/desorption isotherms analysis revealed that TiO_2_–CuO composites exhibit decreasing BET surface areas (119 to 19 m^2^ g^−1^) with higher CuO content, while pore diameters increase (8.6–19.7 nm), confirming mesoporosity (Fig. 6h and i). The optimal TiO_2_ : CuO ratios (7 : 3, 5 : 5, 3 : 7) showed higher surface areas than pure CuO, suggesting enhanced photocatalytic potential due to well-crystalline anatase and CuO phases.

Solvothermal synthesis has emerged as a powerful and versatile technique for fabricating MOx-based composites with precisely tailored microstructures and enhanced functionalities. This method leverages the unique properties of solvents at elevated temperatures and pressures to facilitate controlled chemical reactions and crystal growth. Solvothermal synthesis offers distinct advantages in achieving intricate morphologies, high crystallinity, and homogenous elemental distributions. The solvothermal process typically involves dissolving metal precursors and desired dopants or supporting materials in a suitable solvent, which is then sealed within an autoclave. The autoclave is subsequently heated to a specific temperature, typically ranging from 100 °C to 300 °C, generating autogenous pressure within the sealed vessel. This elevated temperature and pressure environment promotes the dissolution and recrystallization of the precursors, leading to the formation of well-defined MOx nanostructures and their integration with the composite matrix. The choice of solvent plays a crucial role in determining the final product characteristics. Different solvents exhibit varying physicochemical properties, including polarity, viscosity, and coordinating ability, which can influence the reaction kinetics, nucleation, and growth mechanisms. For example, polar solvents like water and alcohols can facilitate the hydrolysis and condensation of metal precursors, while non-polar solvents like toluene and hexane are often employed for the synthesis of organic–inorganic hybrid composites. The versatility of solvothermal synthesis extends to the ability to tailor the morphology and composition of metal oxides composites through careful manipulation of reaction parameters. Adjusting the precursor concentration, reaction temperature, and dwell time can influence particle size, shape, and crystallinity. Furthermore, the introduction of surfactants or capping agents can further modulate the growth process, leading to the formation of hierarchical structures, core–shell morphologies, or other complex architectures. The controlled environment within the autoclave minimizes the introduction of impurities and allows for the incorporation of dopants or supporting materials with high precision. This precise control over composition enables the design of metal oxides composites with tailored electronic properties and enhanced catalytic or adsorptive performance. For instance, incorporating transition metals or other dopants into the metal oxides lattice can modify its electronic structure and improve its catalytic activity towards specific reactions. Similarly, incorporating carbonaceous materials or other high-surface-area supports can enhance the composites' adsorption capacity and facilitate mass transport. For instance, CeO_2_@ZnO core–shell nanostars with a crystalline structure were fabricated *via* hydrothermal and precipitation techniques.^111^ Their crystallinity and nanoscale dimensions were verified by XRD analysis. AFM analysis revealed CeO_2_@ZnO core–shell nanostructures exhibit a nanosized, star-like morphology with high surface roughness (91.64 nm over 2 × 2 μm), enhancing organic molecule adsorption to promote photocatalytic activity, Fig. 6j.^111^ In a separate study, TiO_2_ nanosheets were directly grown on CaTiO_3_ surfaces using a hydrothermal process.^113^ Furthermore, a hydrothermal approach in an ethanol/water solution was employed to load TiO_2_-functionalized graphene oxide onto TiO_2_ nanoparticles.^114^ In addition, a hydrothermal method was used to synthesize a mesoporous CeO_2_/rGO nanocomposites with a surface area of 100.129 m^2^ g^−1^.^115^ In a separate study, a surfactant-assisted hydrothermal approach was used to synthesize multiple layers of a coral-like shaped MgO/g-C_3_N_4_.^116^

Sonochemical synthesis, utilizing the unique effects of acoustic cavitation, has emerged as a powerful technique for fabricating MOx-based composites with tailored properties for advanced environmental applications.^21^ The rapid formation and collapse of microbubbles in a liquid medium under ultrasonic irradiation generate localized hotspots characterized by extreme temperatures and pressures.^21^ These transient, localized conditions promote rapid nucleation and growth, leading to the formation of highly crystalline nanostructures with controlled morphology and enhanced surface area. Furthermore, the intense microstreaming and shockwaves generated by cavitation enhance mass transfer and facilitate uniform dispersion of the metal oxides component within the composite matrix. Specifically, sonochemical methods offer several distinct advantages such as precise control over particle size and morphology, enhanced surface area and porosity, uniform dispersion and intimate interfacial contact, and activation of catalysts and enhanced catalytic activity.^117^ For instance, A CoO–ZnO nanocomposite was synthesized using sonochemical co-precipitation.^118^ SEM micrographs revealed the clumping of spherical particles, which was attributed to the differing magnetic properties of the composite materials.

Sol–gel processing offers a versatile and powerful route for synthesizing MOx-based composites with tailored microstructures and enhanced functionalities for environmental applications. This method leverages the controlled hydrolysis and condensation of metal alkoxides or metal salts in a solution, ultimately leading to the formation of a gel network. This gel, upon subsequent drying and calcination, yields the desired MOx composite. The inherent advantages of the sol–gel method lie in its ability to achieve high purity, homogeneity, and precise control over composition at relatively low temperatures compared to solid-state methods. Moreover, the sol–gel process allows for facile incorporation of dopants and the creation of multi-component composites with intricate architectures. The structural and textural properties of the final metal oxides composite are significantly influenced by several key parameters within the sol–gel process. These include the choice of precursors, solvent, catalyst, water-to-alkoxide ratio, aging time, drying conditions, and calcination temperature. Manipulating these parameters allows for fine-tuning of pore size distribution, particle size, crystallinity, and surface area, which are critical factors in determining the material's performance in applications such as catalysis and adsorption.

In summary, the fabrication of metal oxide (MOx)-based composites for environmental applications demands a strategic balance between synthesis scalability, structural precision, and functional performance. *Ex situ* synthesis approaches, while enabling modular design of pre-optimized components, often struggle with interfacial incompatibility and uneven nanoparticle distribution. *In situ* synthesis approaches, *e.g.*, sol–gel, co-precipitation, and others, excel in achieving homogeneous dispersion and strong interfacial interactions, which are crucial for catalytic and electronic properties, but face challenges in controlling agglomeration during scale-up. Co-precipitation remains a versatile, low-cost route for tailoring crystallinity and defect chemistry, yet its reliance on precise pH and temperature control limits reproducibility. Microwave-assisted synthesis offers rapid and energy-efficient crystallization with fine morphological control but requires optimization of radiation parameters to prevent uneven heating in complex composites. Solvothermal methods provide unparalleled microstructural precision and crystallinity but are constrained by high-pressure/temperature conditions and solvent selection trade-offs. Sonochemical routes promote uniform dispersion *via* cavitational effects but lack scalability, while sol–gel processing enables atomic-level homogeneity at the expense of prolonged processing times. A critical challenge across all methods lies in reconciling the trade-off between achieving nanoscale precision, *e.g.*, defect engineering, core–shell architecture, and maintaining cost-effective and eco-friendly scalability. Additionally, the integration of characterization techniques (*e.g.*, HRTEM, XPS, BET) is indispensable for validating structure–property relationships. Future efforts should prioritize hybrid fabrication strategies, such as microwave-solvothermal or sonochemical co-precipitation, to synergize the advantages of individual methods while mitigating their limitations. Additionally, advancing *in situ* characterization during synthesis and adopting machine learning for parameter optimization could accelerate the development of MOx composites tailored for real-world environmental remediation.

### 3.2 Strategies to enhance photocatalytic performance of metal oxide composites

Extensive research has been conducted to enhance the photocatalytic performance of pristine metal oxides. Strategies such as constructing heterojunctions, doping with other elements, as well as hybridizing with carbon-based nanomaterials, have been explored.^47,119^

#### 3.2.1 Formation of heterojunctions between metal oxides and other semiconductors

**Fig. 7:**
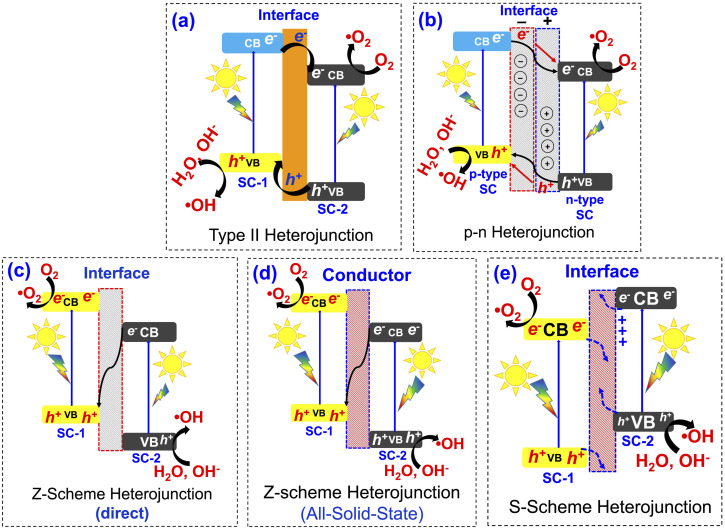
Various photocatalyst heterojunction interfaces: (a) Type-II heterojunction, (b) P–N heterojunction, (c) direct Z-scheme heterojunction, (d) all-solid-state Z-scheme heterojunction, and (e) S-scheme heterojunction, reprinted with the permission of ref. 121, copyright 2025, Elsevier.

Heterojunctions in metal oxide-based composites represent a sophisticated engineering strategy to enhance materials' properties by leveraging the interfacial interactions between dissimilar metal oxides.^120^ These heterojunctions are formed when two distinct metal oxides, such as TiO_2_, ZnO, WO_3_, or CuO, are combined, creating a boundary where their differing electronic structures interact. The interface between the two dissimilar metal oxides creates a built-in electric field that facilitates charge separation and transfer, leading to improved efficiency. Several types of heterojunctions can be engineered depending on the relative band edge positions of the constituent metal oxides including Type II, p–n junctions, Z-scheme, and S-scheme, as shown in Fig. 7.^121–125^ Each of these heterojunction designs plays a pivotal role in improving charge separation and transfer efficiency, thereby facilitating effective redox reactions under visible light illumination.^43,46,126,127^

**Fig. 8:**
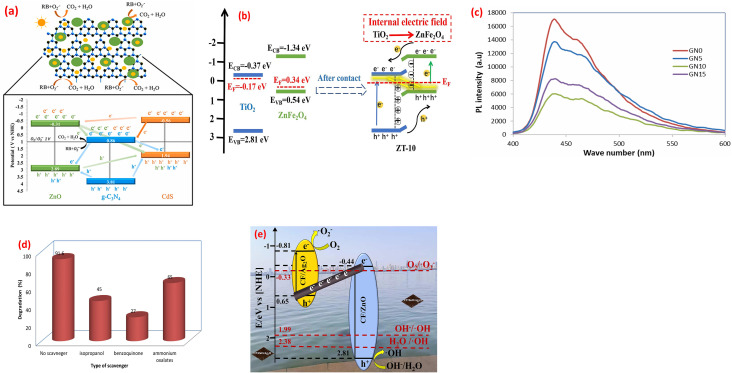
(a) Proposed mechanism of the photocatalytic degradation of RhB using the ternary nanocomposite CdS@ZnO/g-C_3_N_4_, reprinted with the permission of ref. 129, copyright 2025, Elsevier; (b) the bands alignment of ZnFe_2_O_4_, TiO_2_, and ZT-10 nanocomposite, reprinted with the permission of ref. 130, copyright 2025, Elsevier; (c) PL spectra of GN0, GN5, GN10 and GN15 nanocomposite samples, (d) effect of scavengers of degradation of MB on GN10, reprinted with the permission of ref. 122, copyright 2025, Elsevier; and (e) Z-scheme mechanism for photocatalysis of TC by CF/ZnO/Ag_2_O photocatalysts; reprinted with the permission of ref. 131, copyright 2025, Elsevier.

The Type-II heterojunction is a foundational photocatalytic architecture designed to enhance charge separation by leveraging staggered band alignment between two semiconductors. This design is widely used in applications where rapid charge transport outweighs the need for extreme reduction or oxidation capabilities. In a Type-II heterojunction, two semiconductors with offset band structures are combined. Semiconductor A (SC1) has a higher conduction band (CB) and valence band (VB) than Semiconductor B (SC2). When these materials form an intimate interface, their Fermi levels equilibrate, inducing band bending and creating a built-in electric field at the junction. This electric field drives directional charge transfer electrons in SC1's CB migrate to SC2's CB (lower energy), and goes in SC2's VB transfer to SC1's VB (higher energy). For instance, thin TiO_2_/WO_3_·H_2_O layers were created using supercritical CO_2_. This combo splits light-made electrons and holes faster, making reactions quicker. Sunlight excites both materials, creating electrons and holes. Electrons move from TiO_2_ to WO_3_·H_2_O, while holes go to TiO_2_, keeping them apart. Electrochemical impedance spectroscopic tests (EIS) showed the combo moves charges easier than pure parts, lowering resistance.^128^ Further, a ternary CdS QDs, ZnO and g-C_3_N_4_ nanocomposite was synthesized.^129^ The boosted performance of this nanocomposite likely stems from the optimal alignment of electronic energy bands among the three components, which forms combined Z-Scheme and Type-II heterojunctions. These heterojunctions improve charge transfer efficiency, explaining why the CdS@ZnO/g-C_3_N_4_ nanocomposite outperforms single or dual-component systems in photocatalytic activity, as shown in Fig. 8a.^129^ Further, the photocatalytic mineralization of rhodamine B dye (RB) by the α-Fe_2_O_3_@NiO primarily relies on ˙OH and photogenerated h^+^, as confirmed by scavenger tests.^132^ Without scavengers, efficiency was 94%, but adding *tert*-butyl alcohol (˙OH scavenger) and EDTA (h^+^ scavenger) reduced efficiency to 77% and 43%, respectively, highlighting their critical roles. Under UV light, the nanocomposite adsorbs RB dye, initiating charge separation: electrons (e^−^) move to NiO's CB, while holes remain in α-Fe_2_O_3_'s VB. The band alignment, α-Fe_2_O_3_'s VB is more negative (higher energy) than NiO's VB, and NiO's CB is more positive (lower energy) than α-Fe_2_O_3_'s CB, thereby enabling efficient charge transfer. Electrons from α-Fe_2_O_3_ migrate to NiO, while holes shift to α-Fe_2_O_3_, prolonging carrier lifetimes. Reactive radicals (˙OH, ˙O_2_^−^) form *via* reactions between charge carriers, water, and oxygen, attacking adsorbed dye molecules and driving photodegradation. The band alignment between α-Fe_2_O_3_ and NiO indicates a Type II heterojunction. The staggered band structure allows spatial separation of electrons (accumulating in NiO's CB) and holes (accumulating in α-Fe_2_O_3_'s VB), minimizing recombination and enhancing photocatalytic efficiency.

p–n heterojunctions, formed by the interface between p-type (hole-conducting) and n-type (electron-conducting) metal oxides, are pivotal in designing functional materials with enhanced electronic and optoelectronic properties. The junction arises from the contact of two semiconductors with opposing doping characteristics, such as p-type NiO or CuO and n-type TiO_2_ or ZnO. At the interface, Fermi level equilibration drives electron diffusion from the n-type to the p-type material and hole diffusion in the reverse direction, creating a depletion region and a built-in electric field. This field acts as a driving force for charge separation, enabling efficient extraction of photogenerated carriers without external bias. Turns out, some semiconductors aren't strictly n-type or p-type.^133^ For example, the researchers can be messed with a material's structure to tweak it into p-type or n-type, *e.g.*, if ZnO had oxygen missing (oxygen vacancies), it acted as n-type, but if metal atoms were missing (metal vacancies), it flipped to p-type.^134^ In another instance, TiO_2_ with messed-up titanium (Ti vacancies) acted as a p-type, while regular TiO_2_ stayed n-type semiconductor.^135^ The difference in Fermi levels between the p-type and n-type materials drives the diffusion of majority carriers across the interface. Electrons diffuse from the n-type to the p-type material, leaving behind positively charged ionized donors, while holes diffuse from the p-type to the n-type material, leaving behind negatively charged ionized acceptors. This diffusion creates a space charge region, also known as the depletion region, at the interface. Crucially, the resulting separation of positive and negative charges establishes a built-in electric field directed from the n-type to the p-type material. This field opposes further diffusion of majority carriers, ultimately reaching an equilibrium state. The magnitude and spatial extent of this built-in electric field are determined by the doping concentrations, the permittivities of the materials, and the difference in their work functions. This internal electric field plays a critical role in separating photogenerated electron–hole pairs, driving electrons towards the n-type material and holes towards the p-type material, thereby hindering recombination and enhancing the efficiency of charge collection. The synthesized ZnFe_2_O_4_/TiO_2_ (ZT-10) p–n heterojunction enhanced NH_4_^+^–N removal through optimized band alignment and an internal electric field (IEF).^130^ Mott–Schottky analysis confirmed ZnFe_2_O_4_ (p-type, *E*_fb_ = 0.34 eV *vs.* NHE) and TiO_2_ (n-type, *E*_fb_ = −0.17 eV *vs.* NHE) exhibit staggered Type II band edges (ZnFe_2_O_4_: *E*_CB_ = −1.34 eV, *E*_VB_ = 0.54 eV; TiO_2_: *E*_CB_ = −0.37 eV, *E*_VB_ = 2.81 eV). Pre-contact, TiO_2_'s Fermi level (*E*_F_) lay near its conduction band (electron-rich), while ZnFe_2_O_4_'s *E*_F_ resided near its valence band (hole-rich). Upon contact, *E*_F_ equilibration triggered band bending, forming the heterojunction, as shown in Fig. 8b. The IEF spatially separates charges, driving electrons to TiO_2_ and holes to ZnFe_2_O_4_, minimizing recombination and sustaining redox reactions for efficient pollutant degradation.^130^ A nanoflower-structured p–n heterojunction photocatalyst, BiOBr/TiO_2_ (BT-*x*), was synthesized *via* a facile coprecipitation method.^136^ Under visible light, BT-*x* demonstrated superior efficiency in degrading methyl Orange (MO) and gaseous formaldehyde. The p-type BiOBr, with its narrow bandgap, enhances visible-light absorption, while the p–n heterojunction with TiO_2_ promotes enhanced charge carrier separation and transfer, reducing electron–hole recombination. The 3D nanoflower morphology amplifies light utilization through internal scattering, boosting absorption, while also providing abundant exposed active sites for catalytic reactions. The system forms a Type II heterojunction due to the staggered band alignment between p-type BiOBr and n-type TiO_2_.^136^ Furthermore, CuO/ZnO p–n heterojunction nanofibers, were fabricated by integrating p-type CuO with n-type ZnO nanofibers and demonstrated robust efficiency in degrading pyridine from fuel oil.^137^ The enhanced performance stems from boosted-light harvesting and robust separation phenomena. Mechanistic studies revealed that h^+^ primarily drive the formation of reactive intermediates, *e.g.*, ˙O_2_^−^, facilitating pyridine's complete mineralization. The nanofiber structure further optimizes light utilization and charge transfer pathways.^137^ A mesoporous rod-shaped ZnO/CuO/CeO_2_ n–p–n heterojunction was fabricated using a two-step co-precipitation method for photocatalytic applications.^138^ Characterization *via* XRD, FTIR, UV-vis, and SEM confirmed its structure, with interfaces between ZnO (n-type), CuO (p-type), and CeO_2_ (n-type), extending light absorption to 800 nm. Enhanced performance stemmed from broad light absorption, optimized band alignment, and efficient charge carrier separation/transfer. The mesoporous rod morphology improved light harvesting and active site exposure, while the n–p–n configuration facilitated directional charge flow.^138^

Z-scheme heterojunctions represent a promising architecture for enhancing the performance of metal oxide-based photocatalysts by mimicking the natural photosynthesis process. These systems can be categorized into two main types: mediated and direct Z-scheme photoreaction systems, each exhibiting unique charge transfer mechanisms and electric field dynamics. In mediated Z-schemes, a redox couple or a solid-state electron shuttle facilitates the recombination of photogenerated electrons from the photoreaction system II, PSII, analogue with holes from the photoreaction system I, PSI, analogue. This indirect recombination, driven by favorable energy level alignments, preserves the high redox potential of the system by leaving behind highly reductive electrons in the PSI analogue and highly oxidative holes in the PSII analogue.^139^ The internal electric field within each semiconductor component directs charge carriers towards the mediator interface, enhancing the recombination process. Mediator selection plays a critical role, with noble metals offering high conductivity but facing cost and stability issues, while redox couples provide a cost-effective alternative but may exhibit slower kinetics. Solid-state mediators, like reduced graphene oxide (rGO), aim to combine the advantages of both. Thus, adding 10% NiO to g-C_3_N_4_ (forming the GN10 nanocomposite) minimized PL peak by 66%, reflecting suppressed electron–hole recombination, as shown in Fig. 8c.^122^ This modification significantly boosted MB dye degradation efficiency from 33% (using pristine g-C_3_N_4_) to 91.6% (using the GN10 nanocomposite). Scavenger experiments revealed a Z-scheme photocatalytic mechanism, Fig. 8d and e, where visible light-induced charge carriers (electrons and holes) were effectively separated. Superoxide radicals (˙O_2_^−^), generated *via* electron transfer to oxygen, were identified as the primary active species driving MB degradation.^122^ Furthermore, the N–ZnO/g-C_3_N_4_ hybrid, fabricated *via* high-calcination, demonstrated superior performance owing to a Z-scheme charge-transfer mechanism.^140^ The band alignment of N–ZnO enables e^−^ in its CB to migrate to the VB of g-C_3_N_4_, minimizing recombination phenomena. This process retains e^−^ in g-C_3_N_4_'s CB and holes in N–ZnO's VB, boosting redox activity. PL studies employing terephthalic acid (TA) emphasized ˙OH radical generation, with the N–ZnO/g-C_3_N_4_ hybrid showing the robust PL peak (∼460 nm), attributed to 2-hydroxyterephthalic acid (a ˙OH adduct). Pristine ZnO failed to generate ˙OH under visible light, while g-C_3_N_4_ alone exhibited poor ˙OH signals owing to secondary reactions of superoxide radicals (˙O_2_^−^) with water. The composite's boosted ˙OH production stems from efficient hole accumulation in N–ZnO's VB, which oxidizes water.^140^ Further, a Z-scheme Ag_2_O/ZnO heterostructure grown directly on a large-area carbon fiber (CF) substrate (CF/ZnO/Ag_2_O) was developed to boost photocatalytic degradation of TC in water.^141^ The carbon fiber cloth enables easy catalyst recovery and scalability for industrial use, while the Z-scheme design boosts separation and catalytic efficiency. Under light, electrons from ZnO's CB migrate *via* the CF to Ag_2_O, where they recombine with holes (h^+^) in Ag_2_O's VB. This creates a solid-state Z-scheme heterojunction on the CF surface, preserving the strong redox potentials of both ZnO (for oxidation) and Ag_2_O (for reduction), thereby improving TC degradation, as shown in Fig. 8e. Furthermore, solid-state Z-scheme PW_12_/Ag/ZnO, was fabricated by integrating ZnO with Keggin-type tungstophosphate (PW_12_) and incorporating silver (Ag) as a conductive mediator.^131^ The ternary hybrid leverages Ag to enable efficient spatial charge separation and directional carrier transfer, differing markedly from binary counterparts (PW_12_/Ag and PW_12_/ZnO). Experimental results reveal that the ternary system alters the charge migration pathway, enhancing photocatalytic performance. Silver bridges ZnO and PW_12_, facilitating electron transfer between the semiconductors while minimizing recombination losses. This design preserves the redox capabilities of both components, a hallmark of Z-scheme systems, and improves visible-light-driven activity.^131^

**Fig. 9:**
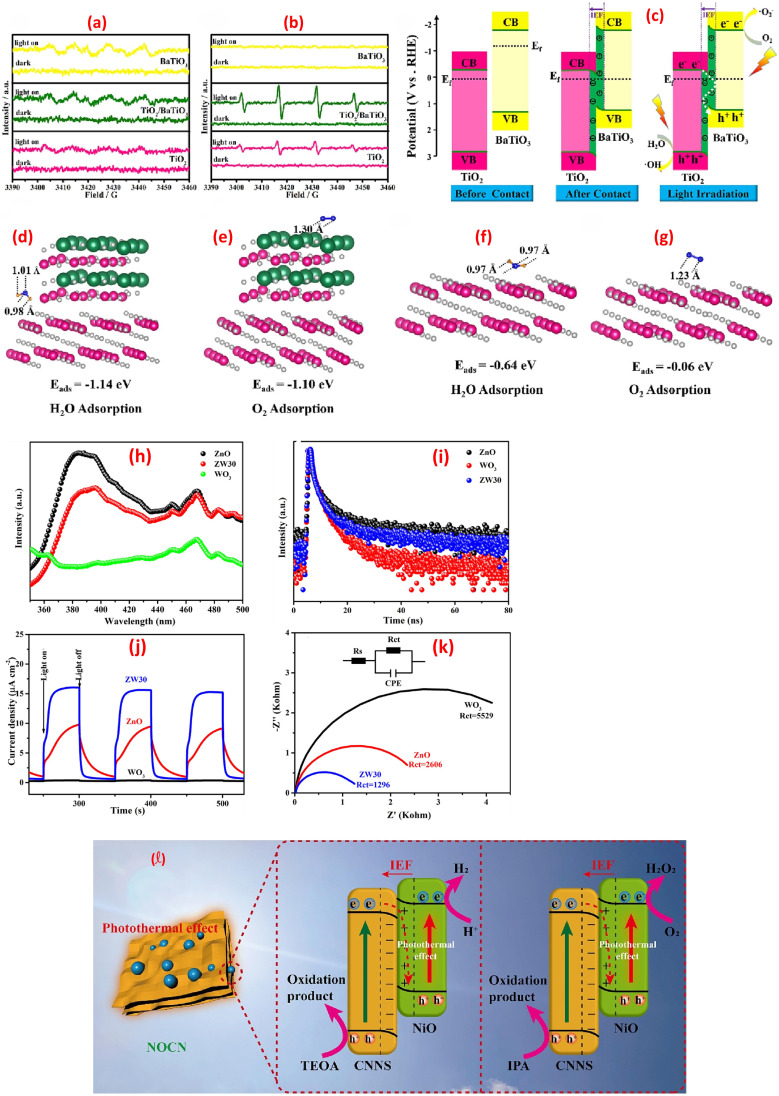
ESR spectra (under dark and light irradiation): (a) DMPO–˙OH (aqueous dispersion), (b) DMPO–˙O_2_^−^ (methanol dispersion). (c) Schematic illustration of the S-scheme charges transfer process on the TiO_2_/BaTiO_3_ heterojunction. (d) H_2_O and (e) O_2_ adsorption on TiO_2_/BaTiO_3_, (f) H_2_O and (g) O_2_ adsorption on TiO_2_, reprinted with the permission of ref. 143, copyright 2025, Elsevier; (h–k) spectra of ZnO, WO_3_, and ZW30, (h) PL spectra, (i) TRPL spectra, (j) transient photocurrent response, and (k) EIS spectra, reprinted with the permission of ref. 144, copyright 2025, Elsevier; (l) photothermal-assisted photocatalytic H_2_ and H_2_O_2_ production *via* NOCN S-scheme heterojunction, reprinted with the permission of ref. 145, copyright 2025, Elsevier.

The S-scheme (Step-scheme) heterojunction is an advanced photocatalytic architecture designed to optimize charge separation while retaining the strong redox potentials of two coupled semiconductors. Unlike traditional Type-II heterojunctions, which sacrifice redox power for charge separation, the S-scheme selectively recombines low-energy charges (electrons from the reduction photocatalyst and holes from the oxidation photocatalyst) while preserving high-energy charges for redox reactions. This mechanism mimics natural photosynthesis but with enhanced efficiency, driven by interfacial electric fields and tailored band alignment. The S-scheme relies on a staggered band structure between two semiconductors: a reduction photocatalyst (RP) with a higher Fermi level (*e.g.*, g-C_3_N_4_, CdS) and an oxidation photocatalyst (OP) with a lower Fermi level (*e.g.*, TiO_2_, WO_3_). When these semiconductors form a heterojunction, their Fermi levels equilibrate, inducing band bending at the interface. This bending generates a built-in electric field that directs charge flow. Electrons from the OP's conduction band (CB) migrate to the RP's CB, and Holes from the RP's valence band (VB) transfer to the OP's VB. The electric field acts as a “charge filter”, promoting recombination of low-energy electrons (OP's CB) and low-energy holes (RP's VB) at the interface. This leaves behind high-energy electrons in the RP's CB (for reduction reactions like H_2_ evolution) and high-energy holes in the OP's VB (for oxidation reactions like O_2_ generation or pollutant degradation). The electric field also spatially separates charges, reducing bulk and surface recombination. For instance, the SnO_2_/SnS_2_ heterojunction exemplifies the S-scheme charge-transfer mechanism, where staggered band alignment preserves the strong oxidative VB of SnO_2_ (∼3.1 eV) and the reductive CB of SnS_2_ (∼−0.2 eV), enabling robust carrier separation while retaining high redox potentials.^142^ Under illumination, interfacial recombination of low-energy carriers (SnO_2_ CB electrons and SnS_2_ VB holes) is driven by the built-in electric field, leaving high-energy holes in SnO_2_'s VB and electrons in SnS_2_'s CB for redox reactions. This selective charge dynamics enhances oxidative ˙OH radical formation (*via* direct H_2_O/OH^−^ oxidation) and reductive 
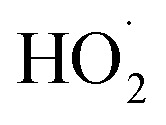
 generation (*via* O_2_ reduction). Synergy between the components further promotes secondary ROS, from 
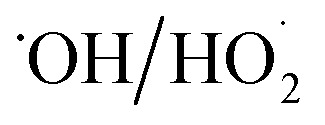
 recombination.^142^ Further, the TiO_2_/BaTiO_3_ heterojunction demonstrates an S-scheme charge-transfer pathway, where a work function disparity (TiO_2_: 6.57 eV, BaTiO_3_: 3.83 eV) drives electron transfer from BaTiO_3_ to TiO_2_, forming an interfacial electric field (IEF), Fig. 9c.^143^ This IEF steers recombination of low-energy carriers (TiO_2_'s CB electrons and BaTiO_3_'s VB holes), preserving high-energy holes in TiO_2_'s VB (1.88 eV) and electrons in BaTiO_3_'s CB for redox reactions. ESR spectra confirm enhanced ˙OH and ˙O_2_^−^ generation, surpassing thermodynamic thresholds for H_2_O/OH^−^ oxidation and O_2_ reduction, Fig. 9a–c. Density Functional Theory (DFT) calculations revealed stronger adsorption of H_2_O (−1.14 eV) and O_2_ (−1.10 eV) on TiO_2_/BaTiO_3_*versus* TiO_2_ (−0.64/−0.06 eV), with elongated O–O/O–H bonds facilitating ROS formation, Fig. 9d–g. Spatial charge localization directs H_2_O adsorption on TiO_2_ (hole-mediated ˙OH) and O_2_ adsorption on BaTiO_3_ (electron-mediated ˙O_2_^−^), bypassing Type-II heterojunction limitations. This synergy ensured efficient toluene mineralization *via* ROS pathways.^143^ A ZnO/WO_3_ S-scheme, fabricated *via* hydrothermal and calcination, exhibited boosted photocatalytic H_2_O_2_ generation *via* a direct two-electron O_2_ reduction pathway.^144^ The interfacial internal electric field in the heterojunction promotes charge migration while preserving electrons with robust reduction capability. PL spectra showed ZW30's lower 390 nm emission *versus* ZnO (Fig. 9h), while WO_3_'s minimal PL intensity reflects its low photoexcitation efficiency. The time-resolved PL (TRPL) spectra revealed ZW30's shorter carrier lifetime (4.4 ns *vs.* ZnO's 5.88 ns), Fig. 9i, indicating reduced recombination. Transient photocurrent (PC) and EIS measurements demonstrated ZW30's rapid photo response, higher current density (15.6 *vs.* 9.4 μA cm^−2^), and lower charge-transfer resistance (1296 Ω), confirming efficient electron transfer, Figure 9k.^144^ A separate investigation demonstrated that black nickel oxide nanoparticles (NiO NPs) were firmly attached to nitrogen-rich graphitic carbon nitride (g-C_3_N_5_) nanosheets (CNNS), forming an S-scheme NOCN heterojunction. This structure enhanced charge carrier separation and redox potential, as shown in Figure 9l.^145^ The study revealed that the NiO NPs boosted light absorption and heat generation capabilities in the NOCN composites compared to their unmodified counterparts.

#### 3.2.2 Metal deposition onto metal oxides photocatalysts

The deposition of noble metals (*e.g.*, Au, Ag, Pt, Pd) on metal oxides introduces transformative effects that address intrinsic limitations of oxide-based photocatalysts. A critical consequence is the suppression of electron–hole recombination *via* the formation of Schottky barriers at the metal/oxide interface. For instance, Pt nanoparticles on TiO_2_ act as electron sinks, trapping photogenerated electrons and prolonging charge carrier lifetimes, which directly enhances redox reaction efficiency in processes like water splitting.^146–148^ Further, the incorporation of Pt into ZnO enhanced photocatalytic CO_2_ reduction by optimizing charge dynamics and light absorption.^149^ The Pt/ZnO nanocomposites with optimal Pt loading (0.75 wt%) formed Schottky junctions, directing electrons from ZnO's conduction band to Pt, suppressing recombination (confirmed by quenched photoluminescence and extended carrier lifetimes ≥2.3 ns). Pt nanoparticles also enabled visible-light absorption *via* Localized Surface Plasmon Resonance (LSPR) and mid-gap states, while acting as catalytic sites that lower activation energies for intermediates (*COOH: −0.28 eV; *CO: −0.15 eV). Photoelectrochemical tests showed a 3.2-fold photocurrent increase and 67% reduced charge-transfer.^149^ In another study, incorporation of Ag onto ZnO significantly boosted photocatalytic activity by establishing a Schottky barrier at the Ag–ZnO interface, which acted as an electron sink to suppress charge recombination.^150^ The Ag/ZnO (S) demonstrated near-complete RhB degradation within 30 min, with kinetic analysis revealing a pseudo-first-order rate constant 2.5 times higher than that exhibited by pristine ZnO, and attributed to prolonged charge carrier lifetimes. Trapping experiments further confirmed that the Schottky-driven charge separation amplified the availability of ˙OH radicals and holes (h^+^), which dominated RhB degradation. Additionally, Ag-NPs improved light harvesting and stability, as evidenced by consistent performance over five cycles.^150^ Furthermore, noble metals such as Au and Ag exhibit surface plasmon resonance (SPR) under visible light, enabling sub-bandgap excitation of wide-bandgap oxides like TiO_2_ or ZnO.^151–153^ This SPR-driven phenomenon generates “hot electrons” that inject into the oxide's conduction band, effectively extending light absorption into the visible spectrum. For example, The Ag–ZnO–CeO_2_ heterostructure was synthesized through a solar-assisted combustion method combined with sequential deposition techniques. Within this composite, Ag exhibited dual functionality: it served as an electron-transfer mediator to promote Z-scheme charge transfer mechanisms and concurrently acted as a plasmonic component, leveraging localized LSPR to enhance optical absorption efficiency within the visible spectrum.^154^ This synergistic integration optimized both interfacial charge separation and light-harvesting capabilities, thereby advancing the material's photocatalytic performance. Furthermore, Ag/ZnO photocatalysts synthesized *via* hydrothermal methods demonstrated enhanced visible-light-driven activity, attributed to the synergistic interplay of Ag nanoparticles' SPR effect and ZnO's semiconductor properties.^155^ The SPR of Ag nanoparticles facilitated intense visible-light absorption, generating energetic hot electrons that injected into ZnO's conduction band, thereby narrowing the effective bandgap (evidenced by UV-vis DRS) and suppressing electron–hole recombination (supported by PL quenching and photocurrent enhancement). This electron transfer mechanism promoted the generation of reactive oxygen species, particularly ˙O_2_^−^ (confirmed by scavenger and EPR experiments), which dominated the degradation of RhB, achieving 96.7% efficiency within 40 min. Furthermore, Ag's SPR-mediated charge separation improved stability, retaining 93.7% activity after five cycles, underscoring its dual role as a plasmonic sensitizer and electron reservoir. Similarly, Au/TiO_2_ nanocomposites synthesized *via* a deposition–precipitation method demonstrated a 36-fold increase in photocatalytic degradation of 2,4-dichlorophenol (2,4-DCP) under visible light compared to pristine TiO_2_.^156^ The SPR effect of Au nanoparticles extended light absorption into the visible spectrum (up to 520 nm) and enabled efficient hot electron injection from Au into TiO_2_'s conduction band, significantly reducing charge recombination (evidenced by enhanced photocurrent and suppressed PL intensity). This electron transfer facilitated the generation of ˙O_2_^−^ radicals (confirmed by scavenger and O_2_-Temperature Programmed Desorption (TPD) experiments), which dominated pollutant degradation, while the Au/TiO_2_ interface maintained exceptional stability over five cycles, underscoring SPR's dual role as a visible-light sensitizer and charge separation promoter.^156^

However, noble metals also introduce challenges, including high costs and nanoparticle aggregation during prolonged reactions. Transition metals (*e.g.*, Cu, Ni, Co, Fe) offer a cost-effective and versatile route to enhance metal oxide photocatalysts, primarily through bandgap engineering and heterojunction formation. Doping transition metals into oxides like ZnO or WO_3_ introduces mid-gap states or modifies d-orbital hybridization, narrowing the bandgap to enhance visible-light absorption.

Combining noble- and transition-metals on metal oxides can yield synergistic effects that surpass the performance of single-metal systems. For example, Ni/Pt co-deposition on TiO_2_ achieved higher CO_2_ yields than either single deposition on TiO_2_.^157^ Moreover, the synergistic deposition of Ni and Pt nanoparticles on TiO_2_ enhanced photocatalytic hydrogen production compared to pure TiO_2_ by reducing the bandgap (*via* dopant-induced quasi-static energy levels), forming a Pt-mediated Schottky junction to accelerate electron transfer, and suppressing charge recombination through Ni–Pt interfacial interactions, while the preferential reduction of Pt^4+^ (over Ni^2+^) and methanol's role as a sacrificial agent further optimized charge separation and proton reduction kinetics.^157^ Similarly, the bimetallic deposition of Au and Cu on Al_2_O_3_ significantly enhanced the catalytic CO oxidation compared to pure Al_2_O_3_ by stabilizing Au nanoparticles against sintering *via* Cu incorporation, optimizing charge transfer between metallic Au and Cu^+^/Cu^2+^ species, and mitigating carbonate-induced deactivation through reactive intermediate formation that preserved active sites under operational conditions.^158^ Further, The co-deposition of Ag and Pt nanoparticles on Ag_3_PO_4_–WO_3_ heterostructures enhanced photocatalytic hydrogen production by synergistically leveraging Ag's plasmonic resonance for broad visible-light absorption, where Pt acted as an electron sink to suppress charge recombination, and the formation of a Schottky junction at the metal-semiconductor interface to facilitate efficient electron transfer, while the Ag_3_PO_4_/WO_3_ heterojunction promoted spatial separation of photogenerated carriers and the green-synthesized Pt–Ag nanoparticles improved dispersion and stability, collectively optimizing redox kinetics for bioethanol reforming.^159^ The co-deposition of Pt and Al on WO_3_ significantly enhanced photocatalytic performance compared to pure WO_3_ by synergistically reducing the bandgap (from 2.36 eV to 1.95 eV) *via* Al-induced lattice distortion and Pt-mediated conduction band modulation, while Pt nanoparticles acted as electron sinks to suppress recombination and oxygen vacancies/Al^3+^ sites improved charge transfer kinetics, enabling 43.61% optical modulation and 85% transmittance recovery *via* efficient Li^+^ intercalation in the porous heterostructure.^160^ The co-deposition of Ag and Cu on TiO_2_ significantly enhanced photocatalytic performance compared to pure TiO_2_ by combining Ag's strong visible plasmonic resonance and Cu's extended light absorption, while optimized photo-deposition time (30 min) ensured controlled nanoparticle growth and uniform dispersion, creating a ternary Ag–Cu–TiO_2_ interface that suppressed charge recombination and accelerated electron transfer, thereby boosting solar-driven degradation efficiency for organic dyes.^161^ Similarly, the co-deposition of Ag and Cu onto ZnO enhanced photocatalytic performance compared to pure ZnO by leveraging Ag's UV plasmonic resonance and Cu's visible-light absorption, while optimized Ag/Cu bimetallic deposition under UV formed combined interfaces that reduced charge recombination and synergized plasmonic effects, achieving 95% degradation; however, under sunlight, monometallic Cu/ZnO (99%) and Ag/ZnO (98%) outperformed due to their direct plasmonic alignment with solar spectra and simpler charge transfer pathways, avoiding the electron loss observed in bimetallic systems.^162^ These hybrid systems also mitigate individual drawbacks: transition metals reduce reliance on costly noble metals, while noble metals compensate for the slower kinetics of transition metal-based catalysts. However, optimizing dual-metal systems require precise control over metal ratios, spatial distribution, and interfacial charge transfer pathways.

#### 3.2.3 Metal and non-metal doping of metal oxides

Doping, the deliberate incorporation of foreign elements into a host lattice, is a pivotal strategy to optimize the photocatalytic efficiency of metal oxides. This process modifies the electronic, structural, and surface properties of these MOx semiconductors, addressing intrinsic limitations. The mechanisms underpinning doping-induced enhancements are multifaceted, involving bandgap engineering, charge carrier dynamics, and surface reactivity modulation. Transition or noble metals (*e.g.*, Fe, Cu, Ag) are introduced substitutionally or interstitially into the MOx lattice. Metal dopants with variable oxidation states create intermediate energy levels within the bandgap, effectively narrowing it and enabling visible-light absorption. Further, metal dopants act as electron or hole traps, delaying recombination. For example, dopants with higher reduction potentials (*e.g.*, Fe^3+^/Fe^2+^: +0.77 V *vs.* SHE) scavenge photogenerated electrons, forming transient Fe^2+^ species, this process prolongs the lifetime of holes in the VB, allowing them to participate in oxidation reactions (*e.g.*, OH˙ generation). However, excessive metal doping risks forming recombination centers or disrupting crystallinity, necessitating optimal doping concentrations (typically 0.1–2 mol%). For instance, the doping of Cu in ZnO promotes activity through multiple mechanisms.^163^ Cu^2+^ ions substitute Zn^2+^ in the lattice, minimizing the optical bandgap (from 3.23 eV in undoped ZnO to 3.08 eV at 20% Cu), enabling visible-light absorption and promoting electron–hole pair generation. Finite-difference time-domain (FDTD) simulations revealed increased electric field intensity due to plasmonic coupling at higher Cu doping, enhancing light utilization.^163^ The 10% Cu-doped sample exhibited optimal performance, attributed to its highest specific surface area (36.97 m^2^ g^−1^, *via* BET measurements), providing abundant active sites for dye adsorption and degradation. Cu-doping also introduces defect states that trap electrons, reducing charge recombination and prolonging carrier lifetimes. Electrical studies confirmed electron hopping as the dominant conduction mechanism, facilitating charge transport. However, excessive doping (20% Cu) increased crystallite size and reduced surface area, diminishing the overall efficiency.^163^ The doping mechanism in iron-doped TiO_2_/ZnO significantly enhanced photocatalytic activity by addressing TiO_2_'s inherent limitations. Iron incorporation narrowed the bandgap of TiO_2_, enabling visible light absorption by introducing intermediate energy states. This extends light utilization and promotes electron excitation. Additionally, Fe doping stabilizes the anatase phase of TiO_2_, delaying its transformation to less active rutile under high calcination temperatures, thereby preserving catalytic efficiency. The dopant also reduces electron–hole recombination by acting as charge traps, improving charge carrier separation. Furthermore, Fe enhances surface properties and active sites, facilitating pollutant adsorption and hydroxyl radical generation. These modifications collectively boost degradation efficiency, as evidenced by 78.99% methyl orange removal under optimized visible light conditions, validated by the study's predictive model.^164^ In another study, Fe^3+^ substitution for Ti^4+^ introduced intermediate energy states, narrowing the bandgap from 3.14 eV (undoped) to 2.84 eV (2.0 wt% Fe), enabling visible-light absorption (400–600 nm).^165^ ESR confirmed Fe^3+^ incorporation, creating a charge-trapping site that minimized recombination phenomena, prolonging carrier lifetimes. Additionally, Fe doping promoted mixed anatase/rutile phases, improving charge separation *via* interphase band alignment. Oxygen vacancies and surface defects from doping increase active sites for reactant adsorption. Despite the reduced BET surface area in Fe/Ti-NTs, these electronic and structural modifications enhanced redox efficiency, achieving 93% methyl orange degradation under UV *vs.* 68% for undoped Ti-NTs.^165^ Further, Sn doping enhanced the photocatalytic activity of WO_3_ by modifying its electronic and optical properties.^166^ Introducing Sn^4+^ into the WO_3_ lattice narrowed the bandgap (from 3.02 eV to 2.80 eV at 4 wt% Sn), enabling visible-light absorption, which broadened solar energy utilization. Sn dopants created defect states that acted as charge-trapping centers, reducing electron–hole (e^−^/h^+^) recombination rates (as evidenced by lower PL intensity), thereby prolonging carrier lifetimes for efficient redox reactions.^166^ Furthermore, Mo doping in WO_3_ nanoparticles enhanced the photocatalytic and electrochemical performance through tailored electronic and structural modifications.^167^ Introducing 5 mol% Mo^6+^ into the WO_3_ lattice narrowed the bandgap from 2.77 eV to 2.49 eV, compared to pure WO_3_, enabling visible-light absorption and reducing the energy barrier for charge excitation. Electrochemically, Mo doping boosted conductivity and charge storage capacity. The specific capacitance increased from 255.6 F g^−1^ (pure WO_3_) to 488.9 F g^−1^ (5% Mo–WO_3_), attributed to improved ion diffusion kinetics and additional redox-active sites from Mo-induced lattice distortions.^167^ The co-doping of Mn and Co in ZnO boosted activity through defect engineering and charge carrier dynamics.^168^ Introducing Mn^2+^ and Co^2+^ ions into the ZnO lattice induced structural defects (*e.g.*, oxygen vacancies) and lattice strain, as confirmed by Raman (520 cm^−1^ peak) and XPS (O 1s spectra). These oxygen vacancies acted as electron traps, suppressing recombination phenomena (evidenced by PL quenching) and prolonging carrier lifetimes for redox reactions. Increased doping concentrations amplified oxygen vacancy density, improving surface reactivity and adsorption of rhodamine B, boosting degradation efficiency (Tables 1 and 2).^168^

**Table 1:** Comparison of different remediation methods

Mechanical methods	Advantages	Disadvantages
Sedimentation	(1) No energy requirement	(1) Ineffective for fine particles (<0.01 mm) and colloids due to low settling rates
	(2) Excellent reproducibility	(2) Space requirements: requires significant area for large-scale operations
	(3) Low operational and maintenance costs due to gravity dependence	(3) Continuous removal of sludge is necessary to prevent quality issues
	(4) Capable of treating large volumes of wastewater efficiently	
Dissolved air flotation (DAF)	(1) Effluent quality: provides superior removal of oils, fats, and suspended solids, yielding high-quality effluent	(1) Significant energy and maintenance costs associated with air generation and system upkeep
	(2) Rapid process: efficient treatment for varying influent qualities and flow rates	(2) Pretreatment requirement: often necessitates pretreatment for optimal performance due to the presence of specific contaminants
	(3) Space efficiency: smaller footprint compared to conventional technologies like sedimentation alone	(3) Requires careful engineering to ensure effective separation
Filtration	(1) Effective removal: capable of removing a wide range of particulates and some pathogens	(1) Clogging: filters can quickly become clogged, necessitating regular maintenance and backwashing
	(2) Flexibility: modular design allows scalability and integration into various systems	(2) Energy use: depending on pressure-driven systems, filtration can be more energy-intensive than passive methods
	(3) Can enhance downstream biological and chemical treatments by eliminating solids	
Screening	(1) Protects downstream equipment from large solids and debris which can cause failures	(1) Only removes larger solids (typically > 1 mm); finer particles may require additional processes
	(2) Ease of operation: generally easy to operate and manage, minimal technical skills required	(2) Regular cleaning: screens must be regularly cleaned to ensure continuous operation (mechanical or manual)
	(3) Cost-effective: significantly reduces the load on subsequent treatment stages	
Centrifugation	(1) Very effective at separating various contaminants, including fine solids; can achieve low solid concentrations in effluent	(1) Requires considerable energy and maintenance, making it expensive to operate
	(2) Capable of rapid processing of sludge and other suspended materials	(2) More complex systems may require skilled operators and regular maintenance to prevent breakdowns
	(3) Smaller equipment footprint compared to traditional settling tanks	(3) Can generate significant noise and vibration during operation

**Physicochemical methods**		
Membrane filtration	(1) Can remove pathogens, bacteria, and solids down to the nanometer range, producing high-quality effluent	(1) High capital and operating costs
	(2) Generates less sludge compared to conventional methods	(2) Membranes are prone to fouling
	(3) Consume small area	
Coagulation and flocculation	(1) High efficiency for fine particles	(1) Chemical use
	(2) Removes metals, colour and turbidity	(2) Needs careful management of chemical dosing to avoid residual chemicals in treated water
	(3) Increases the overall effectiveness of sedimentation and filtration processes	(3) Sludge production
		(4) Multiple process step
Adsorption	(1) Low cost	(1) Low selectivity of adsorbent
	(2) Relatively high performance	(2) Disposal problems
	(3) Design simplicity	(3) Not suitable for high concentration pollutants
	(4) Non-toxic	
	(5) Regeneration of adsorbents is often possible	
Ozonation	(1) Highly effective in disinfection and mineralization of organic pollutants	(1) Ozone is unstable and must be generated on-site
	(2) Produces no harmful residues	(2) Requires careful handling and safety considerations
	(3) No need to alter pH and temperature	(3) In ozone generation, toxicity issues and fire hazards may occur
Advanced oxidation processes (AOPs)	(1) Effective for a wide range of organic contaminants, including those that are resistant to conventional treatment methods	(1) Higher operational costs due to the need for chemicals and specialized equipment
	(2) Can achieve high degradation rates	(2) Potential for toxicity of intermediates
Ion exchange	(1) Possible to regenerate resin	(1) Fouling issue
	(2) Small area requirement	

**Biological methods**		
Aerobic treatment	(1) Simplicity of activity	(1) Foaming problems may arise
	(2) Enhances nitrogen and phosphorus removal	(2) Cost expensive
Anaerobic treatment	(1) Producing biogas	(1) Longer retention times required
	(2) Less environmental pollution	(2) Potential for odor emission
	(3) Reduces sludge volume	
	(4) Can be applied for large capacities	

**Table 2:** Comparisons of representative metal oxides-based heterostructures for organic pollutants' removal^a^

Catalyst	Pollutant	Light source	Time minutes	Removal (%)	Heterojunction type	Ref.
Ce/ZnO	MB	Sun light	120	92.62%	Redox-mediated charge separation	169
TiO_2_-GO	Crystal violet dye	Sun light	150	63		170
NiO/-gC_3_N_4_	MB dye	Sun simulator	90	91	Z-scheme	122
ZnO–C/MnO2	Tetracycline hydrochloride	Halogen lamp (340–800 nm)	92	60	Z-scheme	171 and 172
10% NiO/TiO_2_	Brilliant green	Sunlight	180	87	p–n type	173
TiO_2_/MnO_2_	RhB	75 W metal halide lamp for visible light and 24 W UV lamp for UV light	180	90.50	Z-scheme	174
Bi_2_WO_6_/NiO/Ag	Naphthenic acids	Visible light	180	90	Z-scheme	175
CuO–CdS	TC	300 W Xe lamp	30	86.0	S-scheme	176
CoFe_2_O_4_/g-C_3_N_4_	Enrofloxacin	250 W xenon lamp with a 420 nm cut-off filter	60	89	p–n type	177
ZnCo_2_O_4_/MnO_2_/FeS_2_	MO	500 W halogen lamp	200	96	Z-scheme	178
NiO/Bi_2_WO_6_	Ciprofloxacin	Visible light	90	93	S-scheme	179
Carbon nanosheet/MnO_2_/BiOCl	RhB	300 W Hg lamp	25	97	Z-scheme	180
	MB		40	98		
	TC		30	80		
BiOI/T-ZnOw	RhB	Visible light		97.1	p–n type	181
	OTC			88.0		
ZnO/CeO_2_	RhB	300 W Xe lamp	80	96	Z-scheme	182
B_i2_O_2_CO_3_/ZnO	RhB	Sunlight of 1000 W power	180	97	S-scheme	179
Fe_2_O_3_@e-HNbWO_6_	RhB	300 W Xe lamp	80	99.5	Z-scheme	183
0.1% Ba/ZnO	RhB	Vis-light	60	98.8	Metal doping	184
Ag/ZnO/AgO/TiO_2_	RhB	Xenon lamp (UV-vis) with power of 350 W	100	99.32	Z-scheme	185
MoO_3_/Bi_2_O_4_	RhB	100 W LED lamps	40	99.5	Z-scheme	186
NiO/TiO_2_	MO	UV light (254 nm, 15 W)	30	96.5	p–n type	187
NiFe_2_O_4_/TiO_2_	Congo red	Sunlight irradiation	180	97.0	p–n type	188
AgO/TiO_2_	MB amoxicillin	Sunlight	360	90	p–n type	189
			120	50		
AgI/SnO_2_	Flu	Sunlight of 1000 W power	120	95	Z-scheme	179
TiO_2_/Bi_2_O_3_	Levofloxacin	UV-vis	120	92.7	p–n type	190
AgI/ZnO/WO_3_	MB	100 W tungsten lamp	120	91.5	Double Z-scheme (WO_3_–ZnO and ZnO–AgI)	191

a Pollutants: MB, methylene blue; TC, tetracycline; OTC, oxytetracycline; RhB, rhodamine B; MO, methyl orange; Flu, fluorescence.

On the other hand, non-metals (*e.g.*, N, S, C) replace oxygen anions, altering the VB through orbital hybridization. Nitrogen doping in TiO_2_, for example, raises the VB edge *via* N 2p–O 2p hybridization, narrowing the bandgap and enabling visible-light harvesting. Sulfur's larger ionic radius induces lattice strain, generating oxygen vacancies that serve as electron traps and adsorption sites. These vacancies also promote charge separation by localizing electrons, enhancing surface reactions. Non-metal dopants further influence surface acidity and hydrophilicity, improving reactant adsorption (*e.g.*, H_2_O or O_2_). For instance, N-doping in the ZnO@BiVO_4_ composite induced oxygen vacancy defects in the ZnO component, enhancing charge separation and photocatalytic efficiency, with optimal performance (99.34% tetracycline degradation) achieved at a 20% BiVO_4_ mass ratio under simulated solar light *via* a Type-I heterojunction mechanism.^192^ N-doping in ZnO significantly reduced the bandgap (from 3.12 eV to 2.91 eV), enhancing visible-light absorption and charge separation, which enabled 99% degradation of methylene blue and methyl orange under visible light. Additionally, N-doping introduced oxygen vacancies and mesoporous structures, synergistically boosting supercapacitor performance with a specific capacitance of 762 F g^−1^ in redox-additive electrolytes, driven by improved conductivity and redox-active sites.^193^ N-doping in ZnO reduced its bandgap to 2.99 eV, enhancing visible-light absorption and creating Zn–N bonds that facilitated charge separation *via* a heterojunction with g-C_3_N_4_. This doping introduced oxygen vacancies and increased surface area (147.9 m^2^ g^−1^*vs.* 66.5 m^2^ g^−1^ for pure g-C_3_N_4_), promoting reactive oxygen species (˙OH and ˙O_2_^−^) generation. The 2D–2D interface between N-doped ZnO and g-C_3_N_4_ suppressed electron–hole recombination (evidenced by reduced PL intensity), achieving 96.2% crystal violet and 99.3% brilliant green degradation under visible light.^194^ Nitrogen-doped ZnO supported on biochar photocatalyst derived from Lantana camara leaves (N–ZnO@LBC) exhibited a bandgap reduction from 2.83 eV to 2.78 eV (UV-DRS), enhancing visible-light absorption by introducing mid-gap states, while XRD confirmed lattice expansion due to N substitution at O sites, improving charge separation.^195^ EDX and FTIR spectra validated the N incorporation and hydroxyl group retention, enabling efficient persulfate activation to generate dominant O_2_^−^˙ radicals (scavenger tests).^195^ Furthermore, the 2D/2D heterojunction with biochar suppressed electron–hole recombination, achieving 95.7% MB degradation under visible light and retaining >90% efficiency over 5 cycles, with a treatment cost of US$9.79 per m^3^.^195^ Additionally, sulfur doping in ZnO nanoparticles *via* a low-temperature solvothermal process introduces S 2p-derived mid-gap states, as confirmed by UV-vis and XPS, reduced the band gap (3.24 eV to 3.09 eV) and enabled visible light absorption. This modification enhanced photocatalytic NOx degradation under visible light by 386% due to improved charge separation, while increased surface area (40.7 m^2^ g^−1^*vs.* 29.5 m^2^ g; TEM and BET analysis), and boosted the UV performance by 42%, demonstrating dual structural and electronic optimization.^196^ Moreover, sulfur doping in carbon xerogel/TiO_2_ composites introduced Ti–O–S bonds (as evidenced by XPS data) and mid-gap states, reducing the band gap from 3.0 eV (rutile TiO_2_) to 1.9 eV (as evidenced by DRS data), enabling visible light absorption. This, alongside suppressed TiO_2_ crystallite growth (smaller anatase/rutile phases *via* XRD) and enhanced hydroxyl radical formation under humidity, boosting ethylene photo-oxidation by 25% under visible light and 8% under UV, driven by improved charge separation and sulfur migration from carbon to TiO_2_ during carbonization.^197^ Additionally, sulfur doping in TiO_2_/BiVO_4_ introduced mid-gap states *via* Ti–O–S bonds (confirmed by XPS and Raman), reducing the band gap from 3.22 eV (pure TiO_2_) to 2.10 eV (confirmed by DRS data) and enabled visible-light absorption. Coupled with the TiO_2_/BiVO_4_ heterojunction, sulfur enhanced charge separation (as calculated by DFT simulations) and oxygen vacancy formation, achieving 89.3% dibenzothiophene removal under visible light through improved ˙OH radical generation and interfacial electron transfer.^198^Table 3 shows a comparison of metal deposition and doping approaches.

**Table 3:** Comparison of metal deposition and doping approaches

Feature	Metal deposition	Doping
Mechanism of enhancement	Schottky barrier formation, SPR effect (for specific metals), enhanced light absorption, co-catalytic activity, increased surface area	Modified band structure (band gap narrowing, intra-band gap states), enhanced charge separation (trapping sites), increased charge carrier mobility, modified surface properties
Location of modification	Primarily on the surface of metal oxide	Within the metal oxide lattice
Nature of modification	Formation of a metal-semiconductor interface; physical presence of metal nanoparticles	Introduction of foreign atoms into the metal oxide crystal structure
Light absorption enhancement	Primarily through plasmon resonance (for specific metals) and light scattering; can also indirectly enhance absorption in metal oxide through improved charge separation	Directly modifies the band gap or introduces intra-band gap states, leading to increased absorption in the visible region
Charge separation enhancement	Schottky barrier promotes electron transfer from metal oxide to metal	Dopants act as electron or hole traps, preventing recombination
Charge carrier mobility impact	Can indirectly improve mobility in metal oxide by reducing recombination	Can directly enhance mobility by altering the electronic structure and conductivity of metal oxide
Material stability	Potential for metal leaching or sintering over time, especially under harsh conditions	Generally more stable as dopants are incorporated into the lattice
Examples	Au, Pt, Ag, Pd, Fe deposition on NiO	N, C, Cu, Fe, Al, Li doping of NiO
Disadvantages	Potential for metal leaching, light shielding at high loadings, cost of noble metals	Can be challenging to control dopant concentration and distribution, optimization can be complex

#### 3.2.4 Carbon-based metal oxides nanocomposites

The integration of carbonaceous materials into metal oxide-based photocatalysts resulted in a boosted performance through synergistic mechanisms operating at electronic, optical, and structural levels.^46,199^ Central to this enhancement is the interfacial charge dynamics mediated by the electron sink effect, wherein carbon materials such as graphene, carbon nanotubes, chitosan, and MOFs facilitate directional electron transfer.^46,200^ The disparity in work function between carbon and metal oxides generates a Schottky barrier, establishing a built-in electric field that drives photogenerated electrons from the metal oxide's CB into the carbon matrix. This process mitigates bulk and surface recombination by spatially separating charge carriers. Carbon's delocalized π-electron network further stabilizes photogenerated holes through interactions with oxygen-containing surface groups (*e.g.*, carbonyl, epoxide), thereby suppressing oxidative lattice degradation. The resultant charge separation localizes reduction reactions (*e.g.*, H_2_ evolution) on the carbon phase and oxidation processes (*e.g.*, ˙OH radical formation) on the MOx surface, optimizing redox efficiency (Tables 4 and 5).

**Table 4:** Comparisons of representative metal oxides-based heterostructures for hydrogen production

Catalyst	Synthesis method	Experimental conditions	Heterojunction type	Performance metrics	Advantages	Ref.
NiO–TiO_2_	Hydrothermal calcination	UV-vis light, methanol/water electrolyte	p–n type	23.5 ± 1.2 mmol h^−1^ g^−1^	Enhanced charge separation, stability. However, higher NiO loading but limited by aggregation	201
g-C_3_N_4_/ZnO/Au	Au solution mixed with g-C_3_N_4_/ZnO	Light source: 300 W Xe lamp (UV-vis)	Z-scheme (Au-mediated)	46.46 μmol g^−1^ h^−1^	The Au enhances the absorption of visible light by the LSPR effect, and act as an electron mediator to accelerate the electrons transfer	202
		Electrolyte: 20 mL TEOA + 80 mL H_2_O				
Cu@TiO_2_–Cu_2_O	Solvothermal synthesis	Light source: 300 W Xe lamp (UV-vis)	p–n/Schottky	12.6 mmol g^−1^ h^−1^	Multiple charge transfer channels, enhanced light absorption, highest stability, higher Cu_2_O loading but suffers from aggregation	203
TiO_2_		Electrolyte: 20 vol% methanol in water	Schottky p–n	2.7 mmol g^−1^ h^−1^		
Cu@TiO_2_				5.5 mmol g^−1^ h^−1^		
TiO_2_–Cu_2_O				6.0 mmol g^−1^ h^−1^		
polyaniline/ZnO	Combined sol–gel and oxidative polymerization of aniline	Aqueous solution of methanol (20%). A 300 W Xe lamp	Z-scheme	9.4 mmol h^−1^ g^−1^	Polyaniline is promotes the light absorption and offering an additional electrons that combine with H^+^ to generate H_2_-gas	204
ZnO/g-C_3_N_4_	Solution mixing	Light: 400 W Xe lamp	Z-scheme	1358 μmol g^−1^ h^−1^	Green synthesis (rajma seeds)	205
		Catalyst: 10 mg with Eosin Y dye and 20% TEOA.			Enhanced charge separation (Z-scheme)	
					High surface area (48 m^2^ g^−1^)	
					Visible-light absorption (*E*_g_ = 3.2 eV)	
					Stable for 24 h (HER)	
CeO_2_/CdSe-DETA (CS-2)	Hydrothermal mixing with CdCl_2_, Se, DETA, and N_2_H_4_	Light source: 300 W Xe lamp (*λ* ≥ 420 nm)	S-scheme	3.71 mmol g^−1^ h^−1^	S-scheme pormotes charge separation	206
		Electrolyte: Na_2_S/Na_2_SO_3_ solution			Broad visible-light absorption (up to 746 nm)	
					High stability (6 cycles)	
					Large surface area (∼48 m^2^ g^−1^)	
N-doped CeO_2−*δ*_@ZnIn_2_S_4_	Hydrolysis	300 W xenon lamp, 0.25 M Na_2_SO_3_ + 0.35 M Na_2_S (for H_2_ evolution)	S-scheme	798 μmol g^−1^ h^−1^	EPR confirm that S-scheme is built between ZnIn_2_S_4_ and N-doped CeO_2−*δ*_ which stimulates the separation of the e^−^–h^+^ pairs, and demonstrates exceptional full range operation for photocatalytic HER activity and durability	207
CeO_2_/ZnIn_2_S_4_	Solvothermal (CeO_2_) + oil bath (composite)	3 W UV LEDs (*λ* > 420 nm, 80.0 mW cm^−2^)	S-scheme	69 μmol h^−1^	S-scheme mechanism enhances charge separation	208
		Electrolyte 0.5 M Na_2_SO_3_/Na_2_S			Hollow structure improves light absorption and SSA	
					Internal electric field drives redox reactions	

**Table 5:** Comparisons of representative metal oxides-based heterostructures for carbon dioxide reduction

Composite material	Light source	Reactor type	Co-reactants/sacrificial agents	Product and yield	Main findings	Ref.
Ag QDs/hierarchically porous defective TiO_2_ (Ag/TiO_2_)	300 W xenon lamp (UV-vis, unfiltered)	Catalyst weight: 50 mg	H_2_O (electron donor; holes oxidize H_2_O to ˙OH)	CO: 2.3 μmol g^−1^ (4 h)	Hierarchical pores + defects (Ti^3+^, O vacancies) enhance CO_2_ adsorption and charge separation	209
		Reactor: 80 mL gas-closed quartz reactor		Selectivity: 100% CO (no H_2_ byproduct)	Ag QDs enable SPR for visible light absorption	
		CO_2_ pressure: ambient (purged with CO_2_ for 30 min)				
WO_3_/THFB-COF-Zn (3 : 7 mass ratio)	Simulated visible light (*λ* = 420–800 nm)	Gas–solid phase (quartz reactor)	None/(sacrificial-agent-free)	CO production rate: 54.1 μmol g^−1^ h^−1^ (7× higher than pristine COF)	S-scheme heterojunction: Internal electric field drives e^−^ from WO_3_ CB to THFB-COF-Zn VB. Retains high-potential e^−^ (THFB-COF-Zn CB) for CO_2_ reduction	210
		Catalyst loading: 10 mg dispersed on quartz plate		Selectivity: 100% CO (no H_2_, CH_4_, or HCOOH detected)		
		CO_2_ pressure: pure CO_2_ atmosphere (1 atm)				
		H_2_O source: water vapor (no liquid phase)				
TiO_2_/AC-Ag	300 W Xe lamp (UV-vis)	Catalyst weight: 100 mg	H_2_O (electron donor)	CO: 6× higher than pristine TiO_2_ (relative yield)	AC enhances CO_2_ adsorption (12× higher than pristine TiO_2_) and electron transfer	211
		Reactor: 100 mL quartz reactor			Separate reaction sites: H_2_O oxidation on TiO_2_, CO_2_ reduction on AC.	
		CO_2_ pressure: ambient (continuous CO_2_ bubbling)			Ag SPR extends visible light absorption	
g-C_3_N_4_/WO_3_	300 W Xe lamp (simulated sunlight)	Liquid-phase (gas-closed reactor)	Triethanolamine (TEOA, 2 mL)	CO production rate: 23.0 μmol h^−1^ (2300 μmol h^−1^ g^−1^)	S-scheme charge transfer: e^−^ from WO_3_ CB recombines with h^+^ from g-C_3_N_4_ VB. Retains high-potential e^−^ (g-C_3_N_4_ CB) for CO_2_ reduction	212
		Catalyst loading: 10 mg in 12 mL solvent (H_2_O/acetonitrile/TEOA = 2 : 8 : 2)		CO selectivity: 90.6% (*vs.* H_2_ byproduct)		
		CO_2_ pressure: 1 atm (high-purity CO_2_)				
					Key intermediates: CO_2_*^−^ (1595 cm^−1^), COOH (1380/1628 cm^−1^), CO (2130 cm^−1^) *via in situ* DRIFTS.	
Ag/TiO_2_-*x* nanoparticles-assembly	300 W Xe lamp (simulated sunlight)	Catalyst weight: 50 mg	H_2_O (proton source)	CH_4_: 8.61 μmol g^−1^ h^−1^	Synergy of oxygen vacancies (enhanced light absorption, charge separation) and Ag Schottky junctions (electron trapping)	213
		Reactor: glass dish in sealed reactor		CO: 2.27 μmol g^−1^ h^−1^ (CH_4_ yield 18× higher than TiO_2_)		
		CO_2_ pressure: atmospheric (1 atm)				
α-Fe_2_O_3_/Cu_2_O	300 W xenon arc lamp (*λ* > 400 nm)	0.10 g of photocatalyst; 10 mL of deionized water; CO2 pressure, 0.3 MPa; stainless steel cylindrical reactor	N.A	CO: 1.67 μmol g_cat_^−1^ h^−1^	The tailored heterojunction improved photocatalytic efficiency by optimizing charge pathways, demonstrating how strategic band alignment in oxide composites can amplify redox capabilities for solar fuel synthesis	214
ZnO–Cu_2_O	300 W Xe lamp (UV-vis)	Catalyst weight: 19 mg	H_2_O (0.2 M Na_2_CO_3_, pH 7.4)	CH_4_: 1080 μmol g^−1^ h^−1^	Z-scheme charge separation *via* ZnO–Cu_2_O band alignment	215
		Reactor: 41 mL quartz flask		CO: 1.4 μmol (3 h)	Defect-free surfaces reduce recombination	
		CO_2_ pressure: 2.6 bar (saturation), ambient (reaction)		Selectivity: >99% CH_4_	High surface area (colloidal morphology)	
				QE: 1.5%		
PO_4_^3−^–TiO_2_–Ag_*x*_	300 W Xe lamp (UV-vis)	Catalyst weight: 100 mg	H_2_O (Na_2_CO_3_ solution)	CH_4_: 3.36 μmol g^−1^ h^−1^	PO_4_^3−^ enhances surface hydroxyls, Ti^3^^+^ sites, and CO_2_ adsorption	216
		Reactor: liquid-phase (aqueous suspension)		CO: 0.69 μmol g^−1^ h^−1^ (CH_4_ yield 24× higher than TiO_2_)	Ag nanoparticles form Schottky junctions for charge separation and LSPR.	
		CO_2_ pressure: ambient			Synergy of Ag and PO_4_^3−^ boosts charge transfer and surface reactivity	
Ag/CoO_*x*_-NTO (A/B)	300 W Xe lamp (UV-vis)	Catalyst weight: ∼100 mg (inferred)	H_2_O (Na_2_CO_3_ solution)	CH_4_: 1.37 μmol g^−1^ h^−1^ (NTO-B)	Dual cocatalysts: Ag (electron traps *via* Schottky junctions) and CoOx (hole traps for H_2_O oxidation)	217
		Reactor: liquid-phase (aqueous suspension)		CH_4_: 1.34 μmol g^−1^ h^−1^ (NTO-A)	CoOx supplies H^+^ for CO_2_ reduction, reducing competition with H_2_O	
		CO_2_ pressure: Ambient (Na_2_CO_3_ solution)		(9–12× higher than pristine NTO)	Enhanced charge separation (4.68 s electron lifetime)	
		Material: quartz			Moderate stability (activity decline due to catalyst loss)	
pg-C_3_N_4_/Ag–TiO_2_	300 W Xe lamp (UV-vis)	Catalyst weight: 50 mg	H_2_O (sacrificial agent)	CH_4_: 35.4 μmol g^−1^ h^−1^	S-scheme heterojunction between pg-C_3_N_4_ (porous defective g-C_3_N_4_) and Ag–TiO_2_ enhances charge separation	218
		Reactor: gas-phase (quartz reactor)		CO: 17.3 μmol g^−1^ h^−1^	pg-C_3_N_4_ provides high surface area (59.67 m^2^ g^−1^) and nitrogen defects for CO_2_ adsorption	
		CO_2_ pressure: ambient (purged CO_2_)		Total CO_2_ conversion: 52.7 μmol g^−1^ h^−1^	Ag adjusts TiO_2_'s work function, enabling SPR for visible light absorption and electron transfer	
				AQE: 2.364% (at 420 nm)	Stable for 5 cycles with retained crystallinity	
Ag-cluster/TiO_2_	Xenon lamp (PLS-SXE300, Beijing Perfectlight) with AM 1.5 G filter	Gas-phase reactor: PLR MFPR-I multifunctional Photochemical reactor (150 mL chamber)	No sacrificial agents or photosensitizers used	CH_4_ production rate: 25.25 μmol g^−1^ h^−1^ (*vs.* 0.72 μmol g^−1^ h^−1^ for pristine TiO_2_)	Ag–O hybridization enhances charge transfer and stabilizes intermediates (*COOH, *CHO, *CH_3_O)	219
		Catalyst weight: 5 mg dispersed on quartz dish	Water vapor: 200 μL deionized water added to maintain humidity	CO production rate: 15.92 μmol g^−1^ h^−1^ (*vs.* 3.71 μmol g^−1^ h^−1^ for pristine TiO_2_)	*In situ* DRIFTS confirmed key intermediates (*CO, *CHO) and stronger *CHO adsorption on Ag-cluster/TiO_2_	
		CO_2_ pressure: 105 kPa (99.999% purity)		Electron selectivity for CH_4_: 86% (*vs.* 44% for pristine TiO_2_)	DFT calculations: Ag clusters lower Gibbs free energy for *COOH formation (0.84 eV *vs.* 2.16 eV on TiO_2_) and favor *CO → *CHO over CO desorption	
		Reactor material: quartz (culture dish), sealed chamber with ZnSe windows for *in situ* DRIFTS				
BWO/NiMU_2_	300 W Xe lamp (PLS-SXE300D, full spectrum)	Gas–solid phase (stainless steel chamber with quartz window)	None (sacrificial agent-free)	• CO: 4493 μmol g^−1^ h^−1^	S-scheme charge transfer: e^−^ from BWO CB recombines with h^+^ from NiMU_2_ VB. Ni sites act as CO_2_ adsorption/activation centers	220
		Catalyst loading: 10 mg on sample table + 1 mL H_2_O		• H_2_: 9191 μmol g^−1^ h^−1^	Photothermal synergy: Light drives H_2_O splitting to H^+^. Heat (250 °C) enhances H^+^ spillover to Ni sites	
		CO_2_ pressure: continuous flow (99.999% purity)		CO : H_2_ ratio: 1 : 1.6 to 1 : 2.2 (tunable)		
		Temperature: 250 °C (photothermal conditions)		Selectivity: >95% for syngas		

GO and rGO play pivotal roles in boosting the performance of MOx, with their distinct structural and electronic features enabling tailored functionalities for specific applications. GO is distinguished by its oxygen-rich functional groups, including hydroxyl, carboxyl, and epoxy moieties, which are anchored to its basal planes and edges.^221–225^ These groups widen its bandgap and reduce electrical conductivity compared to rGO, but they significantly enhance adsorption capacity by facilitating π–π interactions and provide reactive sites for redox reactions critical in pollutant degradation.^226,227^ Additionally, the oxygenated structure of GO broadens its light absorption spectrum, albeit with limited efficiency in the visible range. In contrast, rGO is derived from the chemical reduction of GO, a process that reduces oxygen-containing groups and restores a sp^2^-hybridized carbon network closer to pristine graphene. This structural modification drastically improves electrical conductivity and narrows the bandgap, endowing rGO with superior visible-light absorption and charge-carrier mobility.^228^ The mechanisms by which GO and rGO enhance activity are multifaceted. GO's functional groups not only stabilize MOx nanoparticles, preventing agglomeration, but also create diverse reaction pathways through surface-bound radicals and enhanced reactant adsorption. For instance, in MgO@GO composites, GO reduces the bandgap from 2.36 eV (MgO alone) to 1.71 eV, enabling visible-light harvesting and achieving 98% Rhodamine 6G degradation within 15 minutes.^226^ SEM-EDS and UV-vis analyses confirmed GO's role in improving photocurrent response and hydroxyl radical generation, attributed to its high surface area and electron–hole separation efficiency.^226^ Similarly, in CuO/Fe_3_O_4_/GO Z-scheme systems, GO prevented nanoparticle agglomeration, as evidenced by uniform dispersion in SEM/EDS, while its oxygenated sites promote ROS generation. This synergy improved tetracycline degradation to 97.3%, compared to 78.1% for CuO alone, with ˙O_2_^−^ and h^+^ identified as dominant species *via* scavenger tests and EPR analysis.^227^ Conversely, rGO excels in applications requiring rapid charge transfer and minimized recombination. In BiOBr/rGO composites, rGO acted as an electron acceptor, reducing PL intensity by 49% and increasing photocurrent by 2.02× compared to pristine BiOBr.^228^ Its conductive network enhanced adsorption capacity, concentrating pollutants like RhB and TC near active sites, leading to 96% and 73% degradation efficiencies, respectively, under visible light. The dominant pathways, mediated by ˙O_2_^−^ and h^+^, highlight rGO's ability to sustain redox cycles while mitigating charge recombination.^228^

Chitosan serves as a multifunctional scaffold for metal oxide photocatalysts, leveraging its physicochemical properties to enhance catalytic efficiency through synergistic mechanisms. Its amino (–NH_2_) and hydroxyl (–OH) groups play a central role in chelating metal ions (*e.g.*, Ti^4+^, Zn^2+^) during synthesis, facilitating controlled nucleation and growth of nanoparticles (*e.g.*, TiO_2_, ZnO) while preventing agglomeration.^229,230^ This ensures uniform dispersion and maximizes active surface area for redox reactions.^231–235^ During photocatalysis, chitosan mitigates electron–hole recombination by acting as a charge mediator: amino groups trap holes (h^+^), while hydroxyl groups transfer e^−^ to adsorbed oxygen, generating superoxide radicals (O_2_˙^−^).^231–233^ Concurrently, its porous matrix enhances pollutant adsorption *via* electrostatic interactions (*e.g.*, –NH_3_^+^ with anionic dyes) or hydrogen bonding, concentrating pollutants near catalytic sites to promote interfacial electron transfer and reactive oxygen species (ROS) generation (˙OH, O_2_˙^−^).^231,234,235^

The chitosan polymer's hydrophilic nature stabilizes hydroxyl ion (OH^−^) adsorption, favoring ˙OH radical formation through h^+^-mediated oxidation, while its structural integrity reduces metal oxide leaching and enhances durability *via* cross-linking (*e.g.*, with glutaraldehyde).^231–234,236^ Spectroscopic analyses reveal that chitosan induces bandgap narrowing in metal oxides through surface complexation (*e.g.*, Ti–O–C bonds in TiO_2_–chitosan composites) or defect-state formation, enabling visible-light absorption.^231–235^ For instance, in TiO_2_–chitosan hybrids, chitosan immobilized TiO_2_ nanoparticles on substrates, prevented aggregation, and concentrated methyl orange (MO) dye *via* electrostatic adsorption, achieving enhanced interfacial degradation.^231^ FTIR and SEM-EDX analyses confirmed the retention of functional groups and uniform TiO_2_ dispersion, while XRD revealed optimal crystallite sizes (4–18 nm) that balanced light absorption and surface reactivity.^231^ Similarly, in CuO/CS hybrids, chitosan reduced the recombination rate (as confirmed by photoluminescence (PL) data), narrowed the bandgap (as confirmed by DRS data), and enhanced RhB dye mineralization.

Chitosan's multifunctionality extends to complex composites. For instance, in AgZnFe_2_O_4_@CS nanocomposites, CS stabilized MOx nanoparticles, prevented aggregation, and facilitated pollutant adsorption *via* –NH_2_/–OH groups, achieving 81.5% and 82.3% degradation of metronidazole and penicillin G, respectively, under UV light.^234^ FTIR and SEM analyses corroborate chitosan's role in maintaining structural stability and promoting persulfate activation, with SO_4_˙^−^ identified as the dominant radical.^234^ Further, in TiO_2_/CS-biochar composites, chitosan-derived biochar introduced Ti^3+^ and oxygen vacancies during calcination, narrowing TiO_2_'s bandgap and acting as an electron transporter to reduce recombination, resulting in a 30-fold higher RhB degradation rate compared to pristine TiO_2_.^234^ XPS and PL analyses confirmed the enhanced charge separation and defect states.^234^

Further, chitosan enhanced hierarchical architectures such as TiO_2_/CNT/rectorite aerogels, forming a porous lamellar matrix that prevented TiO_2_ aggregation and adsorbed Rhodamine B (RhB) *via* functional groups. The optimized aerogel achieved 95% RhB degradation in 100 minutes, retaining 75% efficiency over three cycles due to structural stability (6). BET and XPS analyses highlighted its high surface area (84.59 m^2^ g^−1^) and TiO_2_ dispersion.^237^ Additionally, in Pt@CS/ZnTiO_3_ systems, chitosan's –NH_2_/–OH groups adsorbed pollutants (*e.g.*, imidacloprid) *via* electrostatic interactions, while its porous matrix synergized with Pt nanoparticles to suppress charge recombination, reducing the bandgap to 2.71 eV (*vs.* ZnTiO_3_'s 2.93 eV).^235^ PL and BET data revealed extended carrier lifetimes and a mesoporous structure (3.07 m^2^ g^−1^), enabling 75% methylene blue (MB) degradation retention over five cycles.^235^

Thus, chitosan transcended the role of passive support, actively participating in adsorption, charge separation, and ROS generation. Its tunable porosity, mechanical stability, and surface functionality enabled tailored integration with metal oxides, establishing it as a critical component in designing robust, solar-driven photocatalytic systems for environmental remediation.

### 3.3 Mechanistic insights into heterostructure performance

**Fig. 10:**
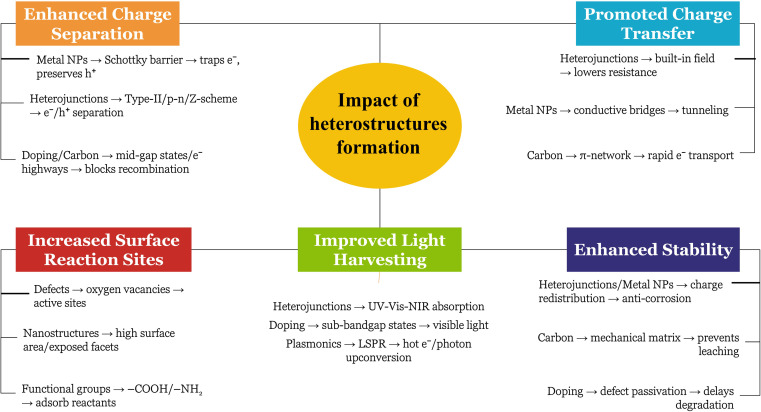
Impact of heterostructure formation on the photocatalytic performance of metal oxides.

As discussed in the previous section, the photocatalytic performance of MOx-based composites is significantly enhanced through heterostructure engineering. This improvement arises from three key mechanisms: optimized charge carrier dynamics, enhanced light absorption efficiency, and amplified surface reactivity. By tailoring the interface between distinct materials, heterostructures mitigate electron–hole recombination, extend the spectral range of photon utilization, and increase active sites for redox reactions. Fig. 10 summarizes the profound influence of heterostructure formation on the photocatalytic activity of metal oxides, demonstrating how strategic material design can elevate their functional efficacy in environmental applications such as pollutant degradation and solar energy conversion.

#### 3.3.1 Enhanced charge separation

The suppression of electron–hole recombination in metal oxide-based photocatalysts is fundamentally governed by the strategic engineering of interfacial electric fields and energy band alignments. Heterojunction architectures, such as Type-II, p–n, Z-scheme, and S-scheme systems, leverage staggered band structures to create built-in electric fields that spatially separate photogenerated charges. In Type-II heterojunctions, the offset between conduction and valence bands of coupled semiconductors drives directional electron transfer to the lower-energy conduction band and hole migration to the higher-energy valence band, minimizing bulk recombination. p–n heterojunctions amplify this effect through Fermi-level equilibration, generating depletion regions that establish strong internal electric fields to segregate charges. Advanced Z- and S-scheme systems further refine charge dynamics by selectively recombining low-energy carriers at interfaces while preserving high-energy electrons and holes with robust redox potentials. These mechanisms are complemented by metal deposition strategies, where noble metals form Schottky barriers at semiconductor interfaces, acting as electron sinks to trap photogenerated electrons and prolong hole lifetimes. Carbon-based composites enhance charge separation by leveraging the high electron mobility of graphene derivatives, which shuttle electrons away from metal oxides, while oxygenated functional groups on carbon matrices stabilize holes. Collectively, these approaches mitigate recombination losses by spatially isolating charges and tailoring energy landscapes to sustain redox-active species for photocatalytic reactions.

#### 3.3.2 Enhanced charge transfer

Efficient charge transfer in metal oxide photocatalysts relies on reducing interfacial resistance and establishing conductive pathways for carrier migration. Heterojunction systems optimize charge transport through band alignment, where the built-in electric field at semiconductor interfaces lowers activation energy for electron and hole movement. For instance, in p–n heterojunctions, the equilibration of Fermi levels induces band bending, creating a directional pathway for majority carriers (electrons in n-type and holes in p-type materials) to traverse the junction with minimal resistance. Metal deposition further accelerates charge transfer by introducing highly conductive metallic nanoparticles that bridge semiconductor components, enabling rapid electron tunneling across interfaces. Doping with transition metals or non-metals modifies lattice conductivity by introducing intermediate energy states or altering orbital hybridization, facilitating band conduction. Carbon-based composites, particularly reduced graphene oxide (rGO), enhance charge mobility through their delocalized π-electron networks, which act as highways for electron transport while mitigating scattering losses. These strategies collectively reduce charge-transfer resistance, as evidenced by EIS, and amplify photocurrent generation, ensuring efficient utilization of photogenerated carriers in redox processes.

#### 3.3.3 Improved light harvesting and band gap engineering

Expanding the optical absorption range of metal oxides requires precise modulation of their electronic structures. Bandgap engineering *via* heterojunction formation combines semiconductors with complementary light-harvesting capabilities, enabling broad-spectrum photon absorption. For example, coupling a wide-bandgap oxide with a narrow-bandgap material extends absorption into the visible or near-infrared regions. Doping introduces mid-gap states through substitutional or interstitial incorporation of foreign elements, narrowing the effective bandgap and enabling sub-bandgap excitation. Non-metal dopants, such as nitrogen or sulfur, hybridize with oxygen 2p orbitals to raise valence band edges, while transition metals create defect states within the bandgap. Plasmonic metal nanoparticles further enhance light harvesting *via* localized surface plasmon resonance (LSPR), where oscillating electrons generate “hot carriers” that inject into adjacent semiconductors, bypassing traditional bandgap limitations. Carbonaceous materials, such as GO or biochar, contribute to light trapping through their inherent optical properties and by inducing defect-mediated absorption in hybrid composites. These synergistic strategies collectively maximize photon utilization, transforming otherwise wasted low-energy photons into catalytically active charge carriers.

#### 3.3.4 Increased surface reaction sites

The photocatalytic efficiency of metal oxides is intrinsically linked to the availability of surface-active sites for reactant adsorption and reaction initiation. Preventing nanoparticle agglomeration through hybridization with carbon matrices or polymeric scaffolds (*e.g.*, chitosan) ensures high surface area and uniform dispersion of catalytic sites. Functional groups on carbonaceous materials (*e.g.*, –COOH, –OH on graphene oxide) or biopolymers (*e.g.*, –NH_2_ on chitosan) enhance adsorption *via* electrostatic interactions, hydrogen bonding, or π–π stacking, concentrating reactants near reactive interfaces. Doping introduces surface defects, such as oxygen vacancies or metal vacancies, which act as trapping centers for charge carriers and adsorption sites for molecular species like H_2_O or O_2_. Hierarchical nano-structuring, such as mesoporous or nanoflower morphologies, amplifies light scattering and exposes high-energy crystal facets, further increasing accessible active sites. These structural and chemical modifications synergistically enhance surface reactivity, ensuring efficient interfacial charge utilization and radical generation.

#### 3.3.5 Enhanced stability

Long-term operational stability of metal oxide photocatalysts is achieved through structural reinforcement and mitigation of photo-corrosion. Heterojunctions stabilize charge carriers by redistributing holes and electrons to less reactive components, reducing oxidative lattice degradation. For instance, in S-scheme systems, selective recombination of low-energy carriers at interfaces minimizes hole accumulation on oxidation-prone semiconductors. Metal deposition forms Schottky barriers that prevent electron backflow, while noble metal coatings shield oxide surfaces from corrosive environments. Carbon matrices and biopolymer scaffolds mechanically stabilize nanoparticles, preventing leaching or aggregation during cyclic operation. Doping passivates surface defects and stabilizes crystal phases, as seen in Fe-doped TiO_2_, which delays anatase-to-rutile phase transitions under thermal stress. These strategies collectively enhance durability, ensuring consistent performance under prolonged irradiation and harsh reaction conditions.

## 4. Photocatalytic application of metal oxide-based composites

### 4.1 Photocatalytic mineralization of pollutants

**Fig. 11:**
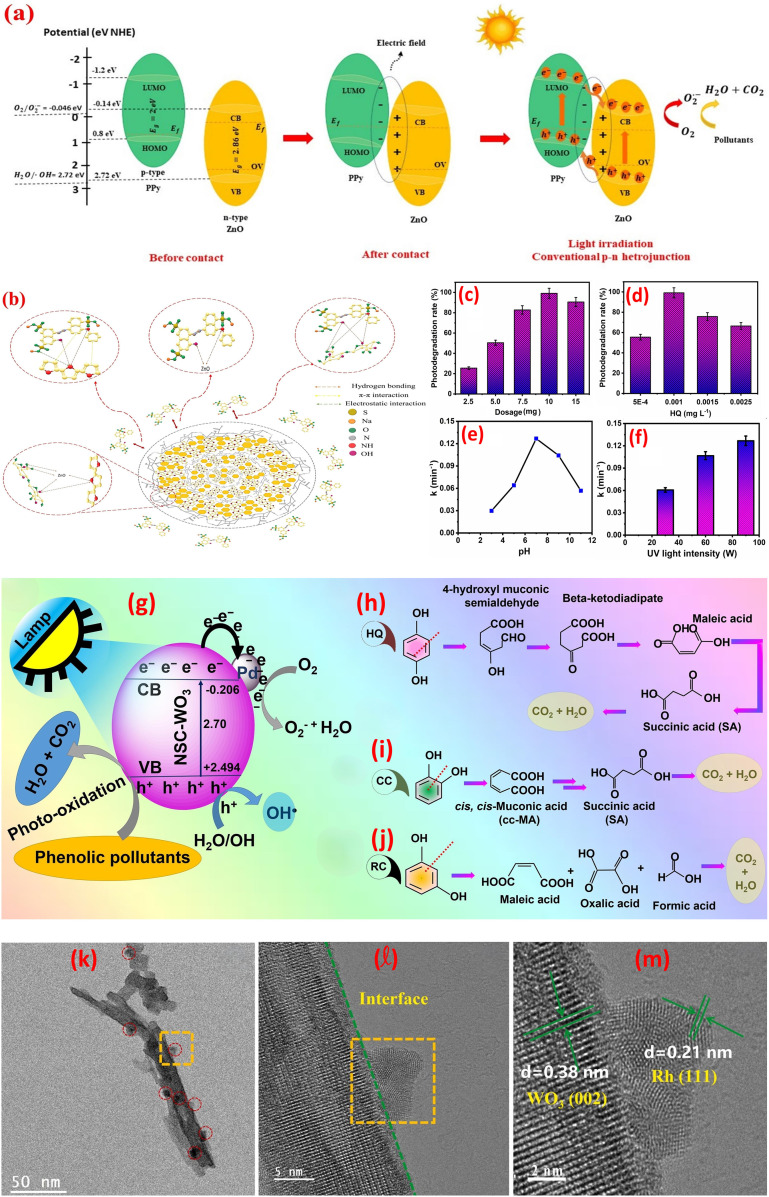
(a and b) Possible photocatalytic degradation and adsorption mechanisms of acid blue 92 (AB92) dye removal by polypyrrole–zinc oxide–sodium alginate (PPy–ZnO–SA) nanocomposite, reprinted with the permission of ref. 239, copyright 2025, Elsevier; (c and d) effects of Pd_2_@NSC-WO_3_ catalyst dose and hydroquinone (HQ) initial concentration on the photocatalytic degradation efficiency of HQ; (e and f) effects of pH and light intensity on the rate constant of HQ degradation; (g) proposed photocatalytic process for efficient photo-oxidation of phenolic pollutants (PPs) using the Pd@NSC-WO_3_ under UV light irradiation and (h–j) the plausible photodegradation pathways for PPs, reprinted with the permission of ref. 240, copyright 2025, Elsevier; (k–m) TEM and HRTEM of 1 wt% Rh/WO_3_ heterostructure photocatalyst, reprinted with the permission of ref. 241, copyright 2025, Elsevier.

The rapid expansion of industrial activities has resulted in a substantial rise in wastewater laden with a wide array of persistent organic pollutants that pose serious risks to human health and environmental sustainability. Heterogeneous photocatalysis, employing metal oxide (MOx) nanomaterials, offers efficient degradation and mineralization pathways of these pollutants. For example, nickel oxide with its favorable band gap and electronic properties, exhibits potential for enhanced photocatalytic activity under UV and visible light irradiation. The generated ROS are the primary agents responsible for the oxidative degradation of organic pollutants. The degradation process involves a complex sequence of reactions, such as hydroxylation, dehydrogenation, and bond cleavage, ultimately leading to the transformation of complex organic molecules into simpler intermediates, resulting eventually in complete mineralization to CO_2_, H_2_O, and inorganic ions. For instance, a ternary NiO/ZnO/g-C_3_N_4_ composite exhibited substantially promoted azo dye degradation compared to its binary combinations and individual constituents.^103^ The enhanced activity, achieving near-complete mineralization of Congo red and methylene blue, is attributed to synergistic impacts including powerful interfacial charge transfer and the role of g-C_3_N_4_ in promoting light harvesting, ROS generation, and charge carrier separation. Similarly, ZnO/NiO/g-C_3_N_4_ photocatalysts, optimized for TC mineralization, exhibited 91.49% removal efficiency within 60 minutes. The mineralization process followed PFO kinetics, with an observed rate constant of 0.05356 min^−1^.^238^ Radical scavenging tests reflected that ˙OH and ˙O_2_^−^ were the dominant reactive oxygen species involved in mineralization of TC. Additionally, an alginate–ZnO–polypyrrole (PPy) hybrid demonstrated effective removal of acid blue 92 (AB92) from aqueous solutions under both UV and visible light irradiation.^239^ The noticed robust removal performance is attributed to the enhanced PPy dye-adsorption and the hindered electron–hole recombination within the ZnO–PPy system, as mechanistically shown in Fig. 11a and b. Furthermore, the Pd@NSC-WO_3_ composite leveraged a dual-function mechanism to efficiently degrade phenolic pollutants (PPs) (1). Under UV light, the engineered heterojunction between Pd and N,S-doped WO_3_ facilitated exceptional charge separation, where Pd acted as an electron reservoir, prolonging the lifetime of photoexcited electrons, while the doped WO_3_ matrix optimized hole mobility (Fig. 11c–j). This synergy amplified radical generation (˙OH and ˙O_2_^−^), which selectively attack aromatic rings *via* various pathways, depending on the pollutant's substituents (*e.g.*, C4/C5 cleavage in catechol). Crucially, the composite's nanoscale architecture (5.1 nm particles) and surface defects enhanced adsorption of PPs and intermediate aliphatic acids, ensuring rapid mineralization to CO_2_/H_2_O. Moreover, Higher catalyst doses enhanced active sites availability, accelerating ROS generation, though excessive loading may hinder light penetration (Fig. 11c). Lower initial PP concentrations favored faster degradation due to reduced competition for reactive species, while acidic pH optimized WO_3_'s surface charge, promoting adsorption of anionic intermediates and stabilizing radicals (Fig. 11d). However, neutral to mild alkaline conditions may enhance hydroxyl radical production *via* OH^−^ oxidation. Furthermore, light intensity directly dictates charge carrier generation, where stronger UV irradiation intensifies electron–hole pair formation, amplifying radical yields and degradation rates.^240^ Further, the bifunctional Rh/WO_3_ catalyst integrated hydrogenation and photocatalytic oxidation to overcome inherent limitations of standalone processes.^240^ Thus, a bifunctional Rh/WO_3_ catalyst was synthesized *via* a liquid-phase reduction method.^241^ TEM and HRTEM analysis (Fig. 11k–m) confirmed the uniform dispersion of Rh nanoparticles (5–10 nm) on WO_3_ nanosheets and revealed distinct lattice fringes (Rh(111): 0.21 nm; WO_3_(002): 0.38 nm), directly visualizing a Schottky heterojunction interface. HAADF-STEM and elemental mapping further validated the atomic-scale integration of Rh with WO_3_, where Rh acted as an electron sink, creating built-in electric fields at the interface to drive charge separation, which is crucial for synergistic hydrogenation and photocatalytic oxidation activity. Additionally, a CeO_2_/BiYO_3_ hybrid was developed for the photocatalytic mineralization of tetracycline (TC).^242^ This material exhibited substantially improved photocatalytic role compared to its individual precursors, BiYO_3_ and CeO_2_, highlighting its promise as an effective photocatalyst. Analysis of band potentials revealed a Type II heterojunction, facilitating efficient charge migration. Moreover, the Ce^3+^/Ce^4+^ redox couple served as an electron trap, further boosting the heterojunction role by minimizing electron–hole recombination. While peak catalytic activity was reported only during the initial cycle, a simple annealing treatment fully restored the catalyst's effectiveness, suggesting excellent reusability of the CeO_2_/BiYO_3_ hybrid.

### 4.2 Hydrogen production

Photocatalytic water splitting offers a direct route to converting solar energy into chemical energy in the form of hydrogen fuel. This process mimics natural photosynthesis but uses a semiconducting photocatalyst material instead of chlorophyll. The process begins when the photocatalyst absorbs photons of light with sufficient energy to excite electrons from the VB to the CB, creating electron–hole pairs. These charge carriers then migrate to the surface of the photocatalyst, where they participate in redox reactions that split water molecules. Crucially, the photocatalyst's band gap and electronic structure must be carefully tuned to straddle the water redox potentials, ensuring that the excited electrons have enough energy to reduce water to hydrogen and the holes can oxidize water to oxygen species. Efficient charge separation is also essential, as recombination of electron–hole pairs diminishes the overall efficiency of the process. At the photocatalyst surface, two distinct half-reactions occur simultaneously. The photogenerated electrons in the conduction band react with water molecules (or protons in acidic conditions) to produce hydrogen gas. This reduction reaction involves the transfer of two electrons to two protons, forming a hydrogen molecule (2H^+^ + 2e^−^ → H_2_). Concurrently, the holes in the valence band drive the oxidation of water molecules, generating oxygen gas and protons. This four-electron oxidation reaction (2H_2_O + 4h^+^ → O_2_ + 4H^+^) is more complex and often kinetically slower than the hydrogen evolution reaction, representing a bottleneck in the overall water splitting process. The efficient extraction of these protons and electrons to the respective reaction sites on the photocatalyst surface is crucial for maximizing the hydrogen production rate. MOx-based composites have emerged as a significant research focus in the field of hydrogen production, owing to their tunable electronic structure and diverse morphologies that can be achieved through strategic synthetic design. For instance, a TiO_2_/NiS core–shell structure was fabricated employing hydrothermal and electrospinning strategies, yielding 655 μmol (g h)^−1^ of hydrogen.^243^ The vertically aligned NiS nanoplates promoted charge migration performance. Furthermore, a hierarchical ZnO/CdS Z-scheme with a microsphere morphology was created.^244^ The optimal CdS loading (30.9%) achieved a hydrogen yield rate of 4134 μmol (g h)^−1^, attributed to improved charge separation and visible light harvesting.^244^ Similarly, a 15 wt% W_18_O_49_/CeO_2_ hybrid exhibited a 1.93-fold increase in hydrogen generation over pristine CeO_2_, resulting from Z-scheme creation.^245^ Furthermore, a WO_3_/TiO_2_/rGO S-scheme heterostructure was developed and achieved a substantially robust hydrogen yield of 245.8 μmol (g h)^−1^, 3.5 times that of pristine TiO_2_.^246^ This improvement stemmed from robust TiO_2_–WO_3_ interactions and a Schottky junction between rGO and TiO_2_, which facilitated charge migration and separation. Moreover, a hydrothermally fabricated TiO_2_ nanosheets on graphene demonstrated a robust hydrogen evolution rate of 736 μmol (h g)^−1^ and a 3.1% quantum efficiency under UV irradiation, with graphene acting as an electron acceptor to enhance charge separation.^247^ Additionally, a GO/CdS/ZnO ternary hybrid achieved a hydrogen evolution rate of 6.511 mmol (g h)^−1^, exceeding the performance of its binary counterparts owing to a narrower bandgap, high surface area, and suppressed electron–hole recombination.^248^ Similarly, an ultrasonically synthesized porous WO_3_/rGO hybrid^249^ exhibited promoted hydrogen evolution (640.5 μmol (g h)^−1^) compared to pristine WO_3_, owing to improved charge diffusion.

### 4.3 Carbon dioxide reduction

**Fig. 12:**
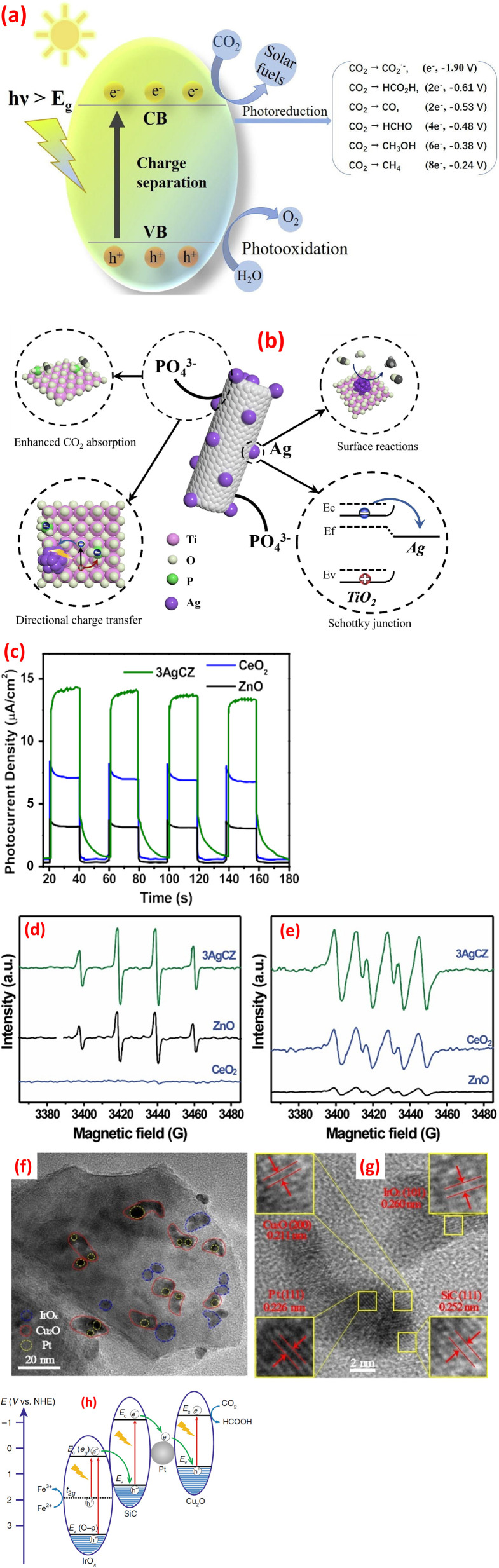
(a) Proposed mechanism of CO_2_ conversion over a MOx-based photocatalyst, reprinted with the permission of ref. 252, copyright 2025, Elsevier; (b) Schematic diagram of photocatalytic CO_2_ reduction on PO_4_^3−^–TiO_2_–Ag_4_, reprinted with the permission of ref. 214, copyright 2025, Elsevier; (c) photocurrent measurements over samples of ZnO, CeO_2_, and 3AgCZ heterostructure; (d and e) EPR spectra of CeO_2_, ZnO, and sample 3AgCZ, (d) DMPO–˙OH and (e) DMPO–˙O_2_^−^, reprinted with the permission of ref. 153 copyright 2025, Elsevier; (f and g) TEM and HRTEM images of Cu_2_O–Pt/SiC/IrOx; and (h) the electron transfer processes in Cu_2_O–Pt/SiC/IrOx under light illumination, reprinted with the permission of ref. 254, copyright 2025, Elsevier.

The photocatalytic reduction of carbon dioxide (CO_2_) using metal oxide-based composites represents a transformative approach to mitigating climate change while producing value-added chemicals. This process hinges on the interplay of light-driven charge carriers, surface chemistry, and reaction engineering to overcome the thermodynamic stability of CO_2_ (Δ*G* = −394 kJ mol^−1^) and reconfigure its molecular structure.^250^ At its core, CO_2_ reduction proceeds through a cascade of proton-coupled electron transfers. Initial steps involve photogenerated electrons (from the composite's conduction band) and protons (derived from water oxidation) destabilizing the linear CO_2_ molecule. The first critical step is a one-electron reduction of CO_2_ to form a bent, activated CO_2_˙^−^ radical intermediate (*CO_2_^−^), followed by C–O bond cleavage and subsequent C–H bond formation.^214,251^ However, the single-electron reduction pathway is thermodynamically prohibitive due to the highly negative redox potential of CO_2_/CO_2_˙^−^ (−1.90 V *vs.* NHE). To circumvent this barrier, proton-coupled multielectron reduction pathways are favored, as they operate at lower redox potentials, enabling feasible activation under ambient or mild conditions (25–100 °C, 1 atm) with UV/visible light irradiation in aqueous or gas-phase reactors (Fig. 12a).^252^ The practical significance of this process lies in its dual role in sustainability and energy innovation. The reduction pathways are highly dependent on the number of electrons transferred. For example, a 2-electron process yields carbon monoxide (CO) or formic acid (HCOOH), while 6–8 electrons are required to generate methane (CH_4_) or methanol (CH_3_OH). These products are dictated by the catalyst's ability to stabilize critical intermediates such as *CO_2_^−^, *COOH, or *CH_3_O. By converting CO_2_, a major greenhouse gas, into fuels (*e.g.*, CH_4_, CH_3_OH) or industrial feedstocks (*e.g.*, CO for syngas), it closes the carbon cycle and reduces reliance on fossil fuels. For instance, the researchers electrochemically deposited Ag nanoparticles within TiO_2_ nanotubes, enhancing SPR-driven CO_2_ reduction *via* amplified light scattering and “hot electron” injection into TiO_2_ (as evidenced by synchronous illumination X-ray photoelectron spectroscopy, SIXPS).^253^ Plasmonic near-field effects reduced charge recombination, yielding 2.3× higher CH_4_ production than surface-loaded Ag. This highlights spatial control of plasmonic metals in oxide scaffolds to synergize light harvesting and charge dynamics for efficient solar fuel synthesis. Further, the researchers developed a novel metal-covalent organic framework heterojunction (WO_3_/THFB-COF-Zn) for efficient CO_2_ photoreduction.^210^ The THFB-COF-Zn framework was synthesized *via* a solvothermal method, by condensing 1,3,5-triazine-2,4,6-tris(4′-hydroxy-5′-formylphenyl)benzene (THFB), ethylene diamine and Zn(ClO_4_)_2_·6H_2_O, whereby the product exhibited enhanced porosity (∼20 Å pore size), and stability in harsh conditions. By integrating WO_3_ nanoparticles, an S-scheme heterojunction was formed and exhibited enhanced charge separation, where electrons move from WO_3_ to THFB-COF-Zn, while holes remain in WO_3_. This design boosted photocatalytic activity, achieving a CO production rate of 54.1 μmol g^−1^ h^−1^ (7× higher than pure THFB-COF-Zn) with nearly 100% selectivity for CO. Key intermediates like *COOH were detected *via in situ* diffuse reflectance Infrared Fourier Transform spectroscopy (DRIFTS), confirming the reaction pathway. The study demonstrates how S-scheme heterojunctions improve CO_2_ conversion efficiency by optimizing charge transfer and light absorption.^210^ In another study, TiO_2_ nanosheets anchored on activated carbon (AC) with Ag nanoparticles (TiO_2_/AC-Ag) enhanced photocatalytic CO_2_ reduction.^211^ Structural analysis confirmed anatase-phase stability, while Ag's SPR effect broadened visible-light absorption and reduced charge recombination (as confirmed by quenched PL intensity). AC's high surface area (427 m^2^ g^−1^) boosted CO_2_ adsorption (12× higher than pristine TiO_2_), and its conductivity facilitated electron–hole separation. Thus, the ternary composite achieved 6× higher CO production than TiO_2_ alone, underscoring synergies between SPR, conductive supports, and adsorption capacity for efficient CO_2_ conversion. Further, engineered α-Fe_2_O_3_/Cu_2_O composites enhanced CO_2_ photoreduction by leveraging interfacial charge dynamics.^214^ An internal electric field at the α-Fe_2_O_3_/Cu_2_O junction directed photogenerated electrons from Fe_2_O_3_'s conduction band to Cu_2_O's valence band, effectively separating charges. This mechanism retained high-energy electrons in Fe_2_O_3_ for CO_2_ reduction while holes in Cu_2_O drove water oxidation to ˙OH and O_2_. The tailored heterojunction improved photocatalytic efficiency by optimizing charge production and separation pathways, demonstrating how strategic band alignment in oxide composites can amplify redox capabilities for solar fuel synthesis. The g-C_3_N_4_/WO_3_ heterostructure, synthesized *via* a two-step process involving calcination, demonstrated enhanced photocatalytic CO_2_ reduction using a H_2_O/acetonitrile/TEOA system under a 300 W xenon lamp. Characterization (XRD, FT-IR, XPS, BET) revealed high crystallinity, meso-porosity (179 m^2^ per g surface area), and effective CO_2_ adsorption (23 cm^3^ per g at 273 K) (16). The composite achieved a CO production rate of 23.0 μmol h^−1^ (90.6% selectivity) with stability over four cycles, outperforming individual g-C_3_N_4_ and WO_3_ due to an S-scheme charge transfer mechanism that improved electron–hole separation. Isotope (^13^CO_2_) and *in situ* DRIFTS analyses confirmed CO_2_ as the carbon source and identified key intermediates (COOH*, CO_2_*^−^), validating the catalytic pathway. This work underscores the potential of crystalline carbon nitride-based heterostructures for efficient solar-driven CO_2_ conversion (16). Furthermore, hierarchically porous Ag/TiO_2_ with Ti^3+^ defects and oxygen vacancies demonstrated enhanced CO_2_ photoreduction to CO under UV-vis light.^209^ Ag quantum dots (≤3 nm) improved visible absorption *via* SPR and facilitated charge separation, while the obtained mesoporous structure with high SSA (68 m^2^ g^−1^) and defects boosted CO_2_ adsorption. The composite achieved 2.3 μmol per g CO yield in 4 h, which is 1.5× higher than pristine TiO_2_, attributed to synergistic electron transfer (Ag → TiO_2_) and defect-mediated activation. Stability tests confirmed minimal Ag leaching, underscoring the role of structural and electronic optimization in sustainable solar fuel synthesis. Further, the synergistic integration of oxygen vacancies and Ag into potholed TiO_2_ nanoparticles *via* solvothermal treatment significantly enhanced photocatalytic CO_2_ reduction by addressing critical bottlenecks in charge dynamics and surface reactivity (Fig. 12b).^213^ Oxygen vacancies narrowed the bandgap (2.7 eV *vs.* 3.1 eV for pristine TiO_2_), extending visible-light absorption and acting as electron traps to suppress recombination (as evidenced by 3× higher CH_4_ yield for TiO_2_-*x* over pure TiO_2_). Ag nanoparticles further amplified charge separation through Schottky junctions, funneling electrons to Ag's SPR-induced “hot spots” and creating electron-rich active sites. The optimized Ag/TiO_2_-*x*-10% composite achieved CH_4_ and CO yields of 8.61 and 2.27 μmol g^−1^ h^−1^, which are 18.3× and 32× higher than TiO_2_, respectively, due to dual pathways: (1) oxygen vacancies promoting CO_2_ adsorption/H_2_O dissociation for proton supply and (2) Ag-driven SPR and Schottky effects enhancing electron density.^213^ Additionally, PO_4_^3−^-modified TiO_2_ nanorods loaded with Ag NPs (PO_4_^3−^–TiO_2_–Ag_*x*_) was synthesized *via* a hydrothermal method, where Ag enhanced visible-light absorption *via* plasmonic effects, while PO_4_^3−^ introduced surface hydroxyl groups and Ti^3+^ sites to improve CO_2_ adsorption and charge separation.^216^ The optimized PO_4_^3−^–TiO_2_–Ag_4_ catalyst achieved a CH_4_ production rate of 3.36 μmol g^−1^ h^−1^, which is 24 times higher than unmodified TiO_2_, due to synergistic effects, where Ag acted as an electron sink, reducing recombination, and PO_4_^3−^ provided Lewis basic sites for CO_2_ activation. *In situ* FTIR revealed *HCOO^−^ and *OCH_3_ as key intermediates in the CO_2_ reduction to CH_4_ pathway, while stability tests confirmed robustness under vacuum reactivation. The approach was further extended to other TiO_2_ phases (*e.g.*, brookite), demonstrating broad applicability for photocatalytic CO_2_ reduction (Fig. 12b).^216^ Another study developed Ag/CoO_*x*_ dual-cocatalyst-modified TiO_2_@Na_2_Ti_3_O_7_ nanoassemblies (NTO).^217^ The Ag/CoO_*x*_ synergy enhanced charge separation, where Ag formed Schottky junctions to trap electrons, while CoO_*x*_ acted as hole sinks for H_2_O oxidation, providing H^+^ for CO_2_ reduction. UV-DRS spectra revealed reduced bandgaps (3.05 → 2.8 eV), and broadened light absorption. Photoelectrochemical tests (EIS, transient photocurrent) confirmed prolonged charge lifetimes (4.68 *vs.* 2.37 s) and lower charge-transfer resistance. Further, an S-scheme heterojunction of Ag-doped TiO_2_ NPs anchored on porous, N-deficient g-C_3_N_4_ (pg-C_3_N_4_) was developed.^218^ The freeze-drying synthesized pg-C_3_N_4_ introduced strategically engineered nitrogen vacancies that dramatically improved CO_2_ adsorption while maintaining structural stability, overcoming a key limitation in defect-engineered photocatalysts. DFT calculations revealed that Ag-doping critically modulated the work function difference between the composite components, creating an interfacial electric field that directed charge separation while preserving strong redox potentials. Another work demonstrated a highly efficient Ag-cluster-modified TiO_2_ photocatalyst (Ag-cluster/TiO_2_) for selective CO_2_ reduction to CH_4_, achieving a remarkable yield of 25.25 μmol g^−1^ h^−1^ with 86% electron selectivity.^219^ The 1 nm-sized Ag clusters, uniformly dispersed onto TiO_2_ microspheres (as confirmed by AC-HAADF-STEM, aberration corrected high-angle annular dark-field scanning transmission electron microscopy), created strong metal–support interactions that significantly enhanced charge separation (25.01 ns carrier lifetime *vs.* 20.48 ns for pure TiO_2_) and CO_2_ adsorption capacity (317 °C desorption peak in CO_2_-temperature-programmed desorption). *In situ* DRIFTS and DFT calculations revealed that interfacial Ag–O hybridization promoted key intermediate (*CHO) formation while suppressing CO desorption, steering the reaction pathway toward CH_4_ production. Furthermore, a ternary Ag–CeO_2_–ZnO hybrid demonstrated enhanced CO_2_ reduction *via* a Z-scheme charge transfer mechanism.^153^ Ag absorbed visible light, generating hot electrons transferred to ZnO/CeO_2_, with suppressed PL emission and higher photocurrent (Fig. 12c) that indicated reduced charge recombination and improved carrier separation. EPR spectra confirmed strong ˙OH and ˙O_2_^−^ radical generation (Fig. 12d and e), which are crucial for redox reactions. The composite achieved CO and CH_4_ production yields of 75.4 and 4 μmol g^−1^ h^−1^, respectively, with 4.47% quantum efficiency at 420 nm, outperforming individual components due to synergistic interfacial electron dynamics and efficient light utilization. Stability tests showed consistent performance over multiple cycles.^153^ Furthermore, a Z-scheme Cu_2_O–Pt/SiC/IrO_*x*_ heterojunction was synthesized *via* sequential photodeposition of Pt NPs (2–4 nm) on SiC, followed by Cu_2_O and IrO_*x*_, as shown in TEM and HTEM images of Fig. 12f and g.^254^ The band alignment between IrO_*x*_, SiC, and Cu_2_O enabled efficient interfacial charge separation, yielding higher photocurrent density compared to Pt/SiC or Cu_2_O–Pt/SiC composites. This enhanced charge dynamics drove CO_2_ photoreduction to HCOOH at 61.5 μmol g^−1^ h^−1^, with Pt acting as a cocatalyst to facilitate electron transfer. The Z-scheme design minimized recombination, optimizing redox capability for solar fuel production (Fig. 12h). Additionally, an S-scheme BWO/NiMU_2_ heterojunction, integrating Bi_2_WO_6_ (BWO) with ultrathin Ni-anchored polymeric carbon nitride (Ni-PCN), demonstrated excellent photothermal CO_2_ reduction to syngas (CO + H_2_) at 250 °C under full-spectrum light.^220^ The heterojunction combined ultrathin PCN nanosheets (2.15 nm, 137.8 m^2^ per g surface area) with atomically dispersed Ni sites, enhancing CO_2_ adsorption (87.45 μmol g^−1^) and charge separation *via* an S-scheme mechanism. This design achieved CO and H_2_ production rates of 4493 and 9191 μmol g^−1^ h^−1^, respectively, with >95% syngas selectivity and stability over 12 hours. Key innovations included Ni single sites (as confirmed by HAADF-STEM data) acting as CO_2_ activation centers, improving electron density (as supported by XPS/DFT data) and reducing charge-transfer resistance (as confirmed by Nyquist plots). EPR/UV-vis/Tauc plots supported the S-scheme charge transfer suppressed recombination, retaining high redox potentials for CO_2_ reduction (*COOH intermediates *via in situ* FT-IR) and H_2_O oxidation. Photothermal synergy (light-driven H^+^ generation and thermal H^+^ diffusion) boosted reaction kinetics, enabling 21× higher H_2_ yield than thermal catalysis alone.^220^

In summary, metal oxide composites are critical for photocatalytic CO_2_ reduction, overcoming challenges like rapid charge recombination and limited light absorption through heterostructures, doping, oxygen vacancy engineering, and integration with conductive layered materials (*e.g.*, graphene, MOFs). Future efforts should focus on combinatorial synthesis guided by DFT and machine learning to optimize band structures, defect densities, and interfacial charge dynamics. Integrating photothermal effects could further enhance kinetics. Moreover, *operando* characterization (*e.g.*, *in situ* XAS, transient absorption spectroscopy) will deepen mechanistic understanding of intermediate stabilization and charge transfer pathways. Selectivity may be controlled through surface modifications, co-catalyst tuning, and adjusting reaction parameters (pH, light intensity and range).

### 4.4 Hydrogen peroxide production

**Fig. 13:**
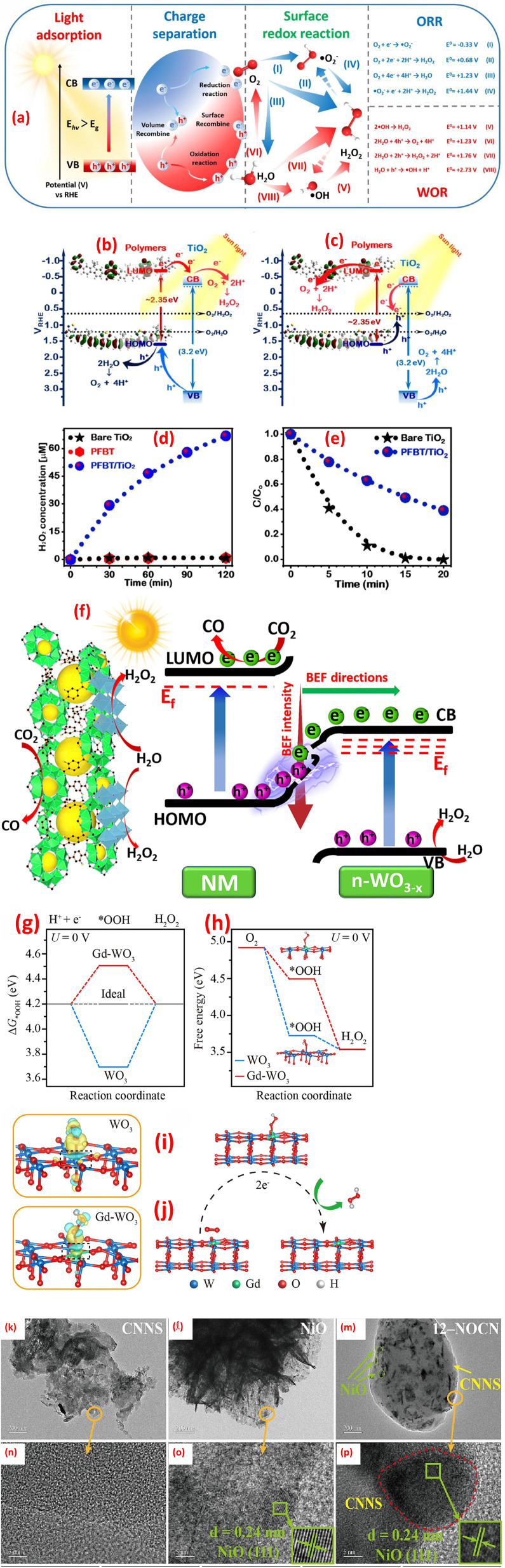
(a) Schematic illustration of the three fundamental processes in photocatalytic H_2_O_2_ production, reprinted with the permission of ref. 256, copyright 2025, Elsevier; (b–e) possible transfer over the organic–inorganic heterojunction following along the band alignment charge transfer pathway I (b), and interfacial charge transfer pathway II (c); (d) photocatalytic generation of H_2_O_2_ of bare TiO_2_, pristine PFBT, and PFBT/TiO_2_ heterostructure without the inclusion of holes scavengers under visible light of 420 nm, 100 mW cm^−2^; (e) the H_2_O_2_ decomposition over bare TiO_2_ and PFBT/TiO_2_ heterostructure under UV-B light of 350 nm, 100 mW cm^−2^, reprinted with the permission of ref. 257, copyright 2025, Elsevier; (f) photocatalytic formation of CO and H_2_O_2_ over NM/*n*-WO_3−*x*_ S-scheme heterojunction, reprinted with the permission of ref. 258, copyright 2025, Elsevier; (g) ΔG_*OOH_ comparison for oxygen reduction on WO_3_*vs.* Gd–WO_3_; (h) 2e^−^ pathway free energy diagram for H_2_O_2_ generation; (i) differential charge densities after *OOH adsorption (yellow/blue: e^−^ – gain/loss); (j) schematic of the 2e^−^ pathway for H_2_O_2_ generation on Gd–WO_3_, reprinted with the permission of ref. 259, copyright 2025, Elsevier; (k–p) TEM and HRTEM images of (k and l) CNNS, (m and n) black NiO NPs and (o and p) 12-NOCN heterostructure, reprinted with the permission of ref. 145, copyright 2025, Elsevier.

Hydrogen peroxide (H_2_O_2_) is a critical chemical with multifaceted applications, ranging from disinfection and environmental remediation to energy storage and green synthesis. Its appeal lies in its potent oxidative capability, eco-friendly decomposition into water and oxygen, and versatility across various sectors.^255^ Several strategies are applied to the production of hydrogen peroxide, such as the anthraquinone (Riedl–Pfleiderer) process, electrolytic processes, byproduct recovery, methane oxidation, *etc.* However, conventional production methods, such as the anthraquinone process, are plagued by high energy demands, toxic intermediates, and complex infrastructure, driving the need for sustainable alternatives.^256^ Photocatalytic H_2_O_2_ generation using metal oxide composites has emerged as a promising solution, leveraging solar energy, water, and oxygen to enable decentralized and low-cost synthesis. In photocatalytic systems, H_2_O_2_ is primarily generated through two competing pathways: the oxygen reduction reaction (ORR) and the water oxidation reaction (WOR) (Fig. 13a).^256^ The efficiency of these processes is further dictated by operational conditions.^255^ Neutral or mildly acidic pH environments are optimal to prevent H_2_O_2_ decomposition, while dissolved oxygen concentration directly impacts ORR kinetics. Light intensity and wavelength also play dual roles: while greater irradiance enhances charge carrier generation, it may accelerate H_2_O_2_ photodegradation. A significant hurdle in scaling photocatalytic H_2_O_2_ production is the reliance on sacrificial agents, such as ethanol or EDTA, to scavenge holes and mitigate recombination. While these agents improve yields, they introduce economic and environmental costs, undermining the sustainability of the process. Recent efforts focus on developing self-sufficient metal oxide systems (*e.g.*, BiVO_4_ or α-Fe_2_O_3_) that balance WOR and ORR kinetics without additives. For instance, the strategic interplay of electronic structure and surface hydrophobicity was validated through multi-modal characterization.^257^ As depicted in Fig. 13b and c, the staggered band alignment between poly(9,9-dioctylfluorene-*alt*-benzothiadiazole) (PFBT, CB: −3.55 eV, VB: −5.9 eV) and TiO_2_ (CB: −4.2 eV, VB: +2.7 eV) creates a directional charge cascade: electrons migrate from PFBT's LUMO to TiO_2_'s CB for O_2_ reduction (2e^−^ pathway), while holes shift to PFBT's HOMO, enabling water oxidation without sacrificial agents. This mechanism is amplified by the polymer's high dipole moments, which enhance charge separation efficiency by 68% compared to bare TiO_2_. The heterojunction's performance is quantified in Fig. 13d, where PFBT/TiO_2_ achieves 67 μM H_2_O_2_ under visible light (>420 nm), clearly contrasting with negligible yields from pristine components. Critically, in the difluorinated derivative, PF2FBT (poly(9,9-dioctylfluorine-*alt*-difluorobenzothiadiazole)), the two fluorine atoms induced hydrophobicity (contact angle: 100.6°, Fig. 13e) and reduced H_2_O_2_ adsorption on TiO_2_ by 82%, slashing decomposition rates from 0.14 min^−1^ (bare TiO_2_) to 0.014 min^−1^.^257^ Another study demonstrated how precise oxygen vacancy (OV) modulation in WO_3−*x*_ amplifies the built-in electric field (BEF) in an S-scheme heterojunction, enabling simultaneous CO and H_2_O_2_ production from CO_2_ and water (Fig. 1f). The controlled OV introduction narrowed bandgaps (2.75 → 2.48 eV) and shifted Fermi levels, with *n*-WO_3−*x*_ achieving optimal alignment for BEF maximization. DFT calculations revealed that OVs introduced W-5d defect states, enhancing visible-light absorption and charge separation. The NH_2_-MIL-125(Ti)(NM)/*n*-WO_3−*x*_ heterojunction forms an intimate interface where BEF-driven S-scheme charge transfer (Fig. 13f) directs electrons from WO_3−*x*_'s CB to NM's LUMO for CO_2_ reduction (12.57 μmol per g per h CO) while holes oxidize water to H_2_O_2_ (8.41 μmol g^−1^ h^−1^). *In situ* XPS confirms light-induced electron redistribution, suppressing recombination by 80% and lowering overpotential.^258^ Further study unveils how Gd^3+^ doping in WO_3_ (Gd-WO_3_) strategically enhances both photocatalytic oxidation and H_2_O_2_ production by engineering electronic and surface properties.^259^ The Gd^3+^ substitutes W sites, introducing mid-gap states that extend light absorption to the near-infrared (1100 nm) and narrow the bandgap (2.30 eV *vs.* 2.42 eV for WO_3_). This doping elevates photocurrent sixfold by leveraging Gd's 4f half-filled orbitals, which act as electron reservoirs to accelerate charge separation. Crucially, DFT calculations (Fig. 13g–j) reveal Gd weakens *OOH adsorption (ΔG_*OOH_ = 4.49 eV *vs.* 3.73 eV for WO_3_), favoring a direct 2e^−^ O_2_-reduction pathway for H_2_O_2_ (0.58 mmol L^−1^ g^−1^ h^−1^) over the 1e^−^ route. Furthermore, in constructing a 2D/1D heterostructure, ultrathin BiOBr nanosheets intimately interface with WO_3_ nanorods, forming coherent junctions that minimize charge recombination.^260^ Advanced characterization (XPS, ESR) reveals that interfacial oxygen vacancies (OVs) act as electron reservoirs, facilitating O_2_ adsorption and activation while inducing localized band bending to optimize charge dynamics. Crucially, the Z-scheme charge transfer mechanism preserves the strong redox potentials of both components: electrons in BiOBr's conduction band drive O_2_ reduction *via* a two-electron pathway, while holes in WO_3_'s valence band oxidize H_2_O to ˙OH radicals, enabling dual-channel H_2_O_2_ synthesis. This structural and electronic synergy, combined with extended UV-vis DRS light absorption, yields an exceptional H_2_O_2_ production rate (8.24 mmol L^−1^ in 2 h), outperforming individual components and physical mixtures (6). Another work integrates CuO/Cu(OH)_2_ nanosheets with g-C_3_N_4_ to harness dual redox pathways: reductive (O_2_ → O_2_˙^−^ → H_2_O_2_*via* g-C_3_N_4_ electrons) and oxidative (H_2_O → ˙OH → H_2_O_2_*via* Cu(OH)_2_ holes). Validated by ESR and PL quenching, a Z-scheme charge transfer minimizes recombination, while Cu(OH)_2_'s hydroxyl-rich surface amplifies oxidative radical yields. Crucially, the hierarchical hollow structure boosts light absorption and charge mobility (EIS/photocurrent), achieving a record 1354 μmol per g per h H_2_O_2_, which is 13.6× higher than CuO alone. Moreover, it exhibited remarkable stability (8 h operation, 0396 mg per L Cu leaching that is still within the specified emission standard for metal ions (1 mg L^−1^, GB 25467-2010)), this approach bypasses noble metals and single-path limitations.^261^ Furthermore, S-scheme NiO/C_3_N_5_ heterojunctions (NOCN) were developed through interfacial anchoring of black NiO onto nitrogen-rich g-C_3_N_5_ nanosheets (CNNS), achieving dual photocatalytic H_2_ (112.2 μmol g^−1^ h^−1^) and H_2_O_2_ (91.2 μmol L^−1^ h^−1^) production under visible light.^145^ TEM/HRTEM analysis revealed NiO NPs (∼10–50 nm) uniformly dispersed on ultrathin CNNS, with lattice fringes of 0.24 nm (NiO (111)) and amorphous CNNS regions, confirming coherent interfacial contact critical for charge transfer, as shown in Fig. 13k–p.^145^ The S-scheme mechanism directs electrons from CNNS to recombine with NiO holes, preserving high-potential electrons (NiO CB: −0.88 V) and holes (CNNS VB: +1.44 V) for robust redox reactions. NiO's broad light absorption induces a photothermal effect (76 °C under irradiation), accelerating charge.

## 5. Cost analysis

The economic viability of modified MOx composite photocatalysts demands a rigorous and quantitative cost analysis, extending beyond rudimentary material expenses to encompass the entire life cycle. This analysis must be intricately interwoven with quantifiable performance metrics to justify the economic benefits of enhanced photocatalytic activity. While heterojunction construction, exemplified by TiO_2_ coupled with WO_3_ or CdS, demonstrably improves charge separation and thus photocatalytic efficiency, the associated cost implications necessitate careful and quantitative scrutiny. Utilizing more economical metal oxides like WO_3_ (approximately ∼20–30 $ per kg), Fe_2_O_3_ (∼5–10 $ per kg), or CuO (∼10–20 $ per kg) presents a less significant cost increase compared to sulfides like CdS (approximately ∼50–100 $ per kg) or more complex oxides like BiVO_4_ (∼50–150 $ per kg). However, the true economic impact is best evaluated by normalizing the cost to the active surface area ($ per m^2^), considering the material density and specific surface area, and correlating it with quantifiable performance enhancements. These enhancements, including improved charge carrier lifetimes (quantified in picoseconds to nanoseconds *via* transient absorption spectroscopy or time-resolved photoluminescence), increased photocatalytic reaction rates (expressed as mol s^−1^ m^−2^ or a defined kinetic rate constant), and enhanced quantum efficiencies (apparent quantum yield or formal quantum efficiency, ideally measured across a relevant wavelength range), should be directly compared against the incremental cost per unit area. Such a data-driven approach enables a robust cost-benefit assessment and facilitates direct comparison between different material combinations. Moreover, MOx incorporating non-metal dopants offers a potential avenue for cost-effective performance enhancement. Nitrogen doping, achievable through a simple annealing process in ammonia or nitrogen gas, incurs minimal cost increases (<1%) and can significantly modify the electronic band structure, leading to enhanced visible light absorption. Similarly, carbon doping, utilizing readily available precursors like carbohydrates, can introduce defect sites and improve charge separation. Quantifying the impact of these non-metal dopants on band gap energy (*via* DRS), charge carrier dynamics, and ultimately, photocatalytic activity, is crucial for evaluating their cost-effectiveness. Comparing the cost per unit enhancement (*e.g.*, $ per % increase in quantum yield) for non-metal doping against the cost associated with metal doping or heterojunction formation provides a valuable metric for material selection. Moreover, incorporating co-catalysts, such as earth-abundant metal sulfides like MoS_2_ or NiS, presents an alternative to noble metals like platinum (Pt, approximately ∼30 000 $ per kg) or gold (Au, significantly higher cost). While these earth-abundant co-catalysts might require optimized loading and careful synthesis to achieve comparable performance enhancements, their significantly lower cost makes them attractive candidates for large-scale applications. Quantifying their impact on hydrogen evolution rates (in photocatalytic water splitting), pollutant degradation kinetics, or other relevant performance metrics, alongside a detailed cost breakdown of their synthesis and integration, is essential for evaluating their economic viability. The integration of carbon materials further complicates the cost landscape. High-quality graphene and carbon nanotubes (CNTs), priced between ∼100 and ∼1000 $ per g, significantly impact overall material cost, while activated carbon, at ∼1 $ per kg, provides a more economically viable alternative. Quantifying the performance benefits conferred by these carbon materials, such as increased surface area, enhanced charge transfer efficiency (measured through EIS), and improved photocatalytic activity, against their respective costs and loadings is crucial. Optimizing the loading of expensive materials like graphene and rigorously assessing their long-term stability, including potential leaching under real operational conditions, is paramount for ensuring economic viability.

Synthesis technique critically dictates cost and performance of modified metal oxide photocatalysts. Precise, but expensive, techniques like atomic layer deposition (ALD) and chemical vapor deposition (CVD), >0.10 $ per cm^2^ offer superior control over film properties and heterostructures but face scalability challenges. Conversely, solution-based methods such as coprecipitation, or sol gel approach (<$0.01 per cm^2^) offer cost advantages but compromise precise morphological control. Quantifying energy consumption and comparing structural properties (surface area, size distribution, crystallinity) across methods is crucial for optimizing cost-performance trade-offs. Emerging methods like microwave and sonochemical syntheses warrant investigation for enhanced efficiency and control. Finally, a comprehensive techno-economic analysis must incorporate a detailed life cycle assessment (LCA) and extend beyond simple material costs. This should include precise quantification of raw material costs, synthesis costs (including energy consumption, labor, equipment depreciation, and facility overhead), catalyst lifetime and replacement costs, and end-of-life management, including disposal or recycling costs. Quantifying the cost per unit of treated effluent ($ per m^3^) or product generated ($ per kg or $ per mole), coupled with LCA data on environmental impacts such as CO_2_ emissions, water usage, and potential eco-toxicity, provides a holistic framework for evaluating the true cost and sustainability of these photocatalytic materials. This quantitative, data-driven approach facilitates informed decision-making, optimizing material selection, synthesis methods, and operational parameters for economically viable and environmentally responsible photocatalytic technologies.

## 6. SWOT analysis

**Fig. 14:**
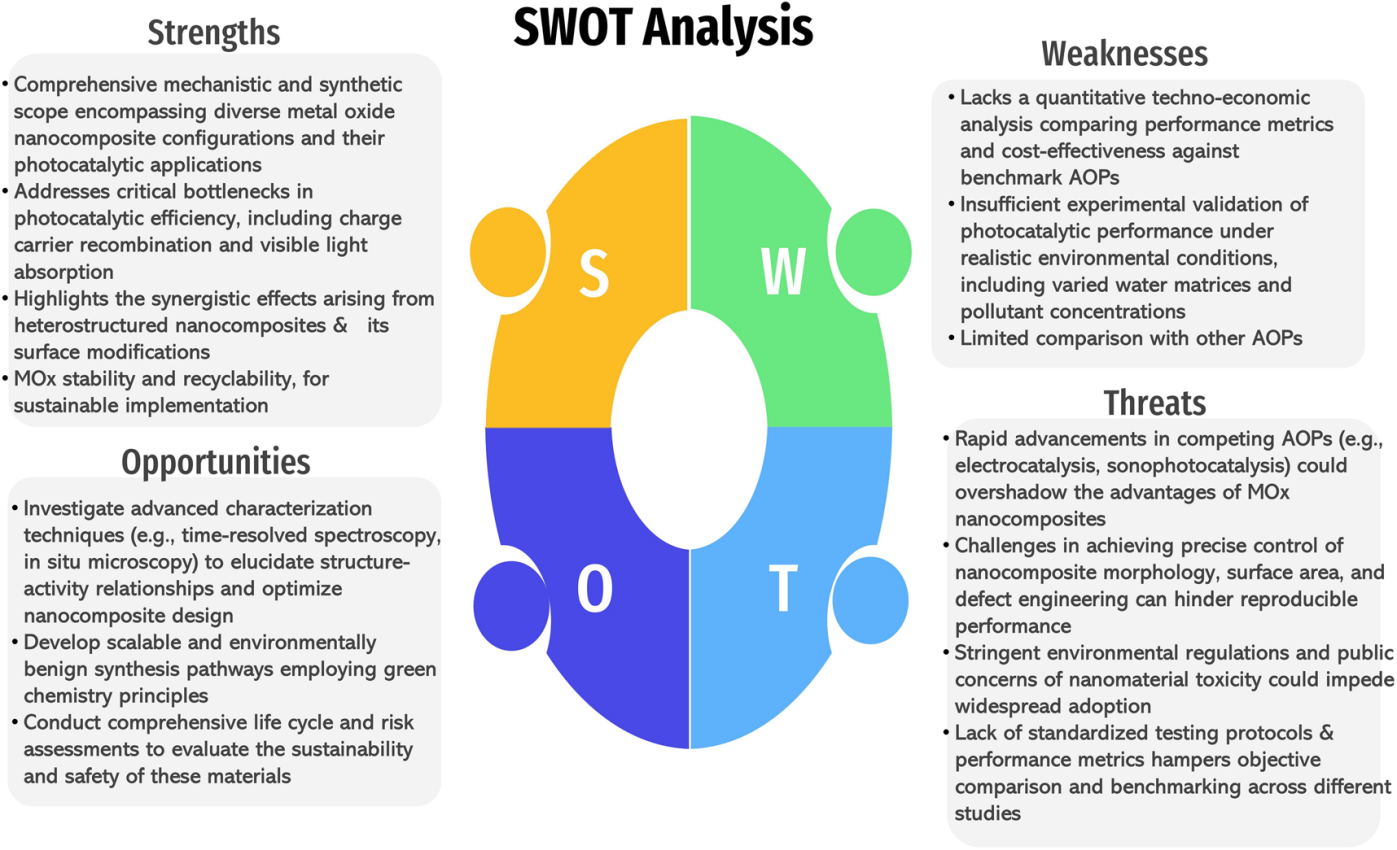
Summary of SWOT analysis of the synthesis, characterization and applications of MOx in photocatalytic processes.

This review explores the promising field of metal oxide nanocomposite photocatalysis for environmental remediation and energy generation. It begins by examining the fundamental mechanisms governing photocatalysis and delves into the synergistic effects achieved by combining MOx with other nanomaterials. This comprehensive scope encompasses a range of topics from conventional wastewater treatment approaches to the cutting-edge advancements in MOx nanocomposite photocatalysis, including material synthesis, mechanistic insights, influencing factors, and future research directions. Furthermore, it addresses critical aspects such as material stability and recyclability, key considerations for practical implementation. The inherent sustainability of this technology, aligning with global environmental goals, is also emphasized. Despite the significant potential, several key areas require further investigation to fully realize the promise of MOx nanocomposite photocatalysis. A crucial gap lies in the need for a rigorous cost–benefit analysis. While the potential for cost-effectiveness is acknowledged, a detailed assessment comparing production costs, material expenses, operational costs (including energy consumption and maintenance), and the economic benefits of resource recovery (*e.g.*, energy generation, reclaimed water) against established technologies is lacking. This comparative analysis is essential for evaluating economic viability and promoting practical implementation. Furthermore, the potential of these materials extends beyond pollutant degradation. Integrating photocatalysis with electrochemistry, for example, offers exciting possibilities for simultaneous pollutant removal and energy harvesting. However, this aspect of photoelectrochemical applications remains relatively unexplored and warrants further investigation. Leveraging advanced characterization techniques, such as TRPL and *in situ* microscopy, can provide deeper insights into the structure–activity relationships governing photocatalytic performance. This knowledge is crucial for optimizing nanocomposite design and maximizing efficiency. Furthermore, the literature lacks actual examples of successful applications that include thorough performance statistics. Showcasing specific case studies, including removal efficiency of target pollutants (heavy metals, or specific organic species) under realistic settings and in varied water matrices, will considerably increase the practical relevance. Quantitative comparisons against existing technologies for these specific cases are particularly valuable. Beyond cost–benefit analysis and practical examples, a comprehensive comparative analysis with other AOPs is warranted. While this review primarily focuses on MOx nanocomposites, a more detailed comparison of their advantages and disadvantages against other AOPs in terms of efficiency, cost, environmental impact, and applicability to different pollutants would provide a more balanced perspective. Similarly, while the potential for energy generation using MOx photocatalysis is reviewed, a deeper exploration of specific strategies, realistic energy yields, and associated technical challenges is needed to fully assess this application. Finally, the review acknowledges the importance of recyclability and stability but needs to expand on other practical implementation challenges. These include reactor design and optimization (light penetration, mass transfer limitations), long-term performance and fouling, safe handling of nanomaterials, potential environmental risks of nanoparticles, and regulatory hurdles for real-world applications. Several opportunities emerge from these identified gaps. Further research should prioritize exploring synergistic effects of specific MOx combinations and optimizing their synthesis methods for enhanced photocatalytic activity. Developing scalable and cost-effective production methods is crucial for practical implementation. Investigating novel applications beyond wastewater treatment and energy generation, such as air purification or CO_2_ reduction, could broaden the technology's impact. Addressing the recyclability and stability challenges, as well as conducting life cycle assessments, are also crucial. Finally, it's important to acknowledge potential threats to the widespread adoption of this technology. Competition from other emerging wastewater treatment and energy generation technologies, challenges in scaling up production while maintaining cost-effectiveness, navigating regulatory requirements, ensuring public acceptance of nanomaterials, and the lack of standardized testing protocols are all potential obstacles that must be addressed. Fig. 14 shows a summary of SWOT analysis of the synthesis, characterization and applications of MOx in photocatalytic processes.

## 7. Conclusion and prospectives

The transformative potential of metal oxide-based composites in photocatalysis lies at the intersection of material innovation, environmental urgency, and energy sustainability. By engineering heterojunctions (*e.g.*, S-scheme, Z-scheme), tailoring defects (oxygen vacancies, dopants), and integrating hybrid matrices (graphene, biochar, polymers), these composites surmount the inherent limitations of pristine MOx, achieving unprecedented charge separation, broad-spectrum light absorption, and robust redox activity. Advanced synthesis methods, from solvothermal crystallization to bio-inspired green chemistry, enable atomic-level control over morphology and interfacial dynamics, directly translating to enhanced photocatalytic efficiency. Yet, critical challenges remain, *e.g.*, long-term stability under harsh operational environments, scalability of nanoscale precision to industrial volumes, and the economic-ecological balance of material production. To propel MOx composites from laboratory curiosities to societal solutions, the following prospective directions demand urgent attention:

(1) Scalable synthesis & green manufacturing: develop scalable and cost-effective methods for synthesizing high-efficacy MOx nanomaterials and fabricating functional systems, while prioritizing earth-abundant elements (*e.g.*, Fe, Zn) and waste-derived precursors (*e.g.*, biomass, slag) for sustainable and environmentally friendly large-scale production.

(2) Durability enhancement: develop more effective and stable MOx-based nanocomposites that can withstand harsh environmental conditions (extreme pH, UV exposure) while retaining their favorable adsorptive and photocatalytic properties over extended life cycles.

(3) Advanced hybrid design: develop ternary/quaternary MOx hybrids with green carbon-based materials to enhance charge dynamics and enhance photocatalytic efficiency. Explore bio-inspired architectures (enzyme-mimetic co-catalysts, cyanobacteria-MOx composites) for selective CO_2_ conversion.

(4) Reactor innovation & process integration: design modular, solar-optimized reactors with light-trapping features (plasmonic waveguides) and integrate MOx systems into hybrid platforms (*e.g.*, photocatalytic membrane reactors) for simultaneous pollutant degradation and resource recovery.

(5) Real-world validation & standards: conduct pilot-scale studies in wastewater/air purification systems. Establish global benchmarks for efficiency (quantum yield) and durability, supported by academia-industry consortia.

(6) Risk mitigation & circular economy: screen for ecotoxicity and design recyclable composites (magnetic cores, pH-responsive materials). Adopt lifecycle assessments (LCAs) to align with zero-waste goals. Future studies should focus on developing mitigation strategies and conducting comprehensive risk assessments to ensure responsible development.

(7) Computer-aided material optimization: leverage machine learning to predict dopant combinations, heterojunctions, and defect densities. Use generative prediction and simulation for novel architecture (*e.g.*, MOx-MOF hybrids).

(8) Operando mechanistic insights: employ synchrotron techniques (XANES) and ultrafast spectroscopy (fs-TAS) to map defect evolution and carrier dynamics, guiding real-time catalyst design. Further, employing advanced techniques, such as TRPL and *in situ* microscopy, can provide deeper insights into structure–activity relationships, guiding optimized MOx nanocomposites design and maximizing photocatalytic efficiency.

(9) Policy & interdisciplinary collaboration: advocate for standardized toxicity protocols and funding models to accelerate industrial adoption of MOx technologies.

(10) Economic viability frameworks: balance efficiency with costs (material, energy) and end-of-life recyclability, ensuring scalability for global environmental applications.

**Fig. 15:**
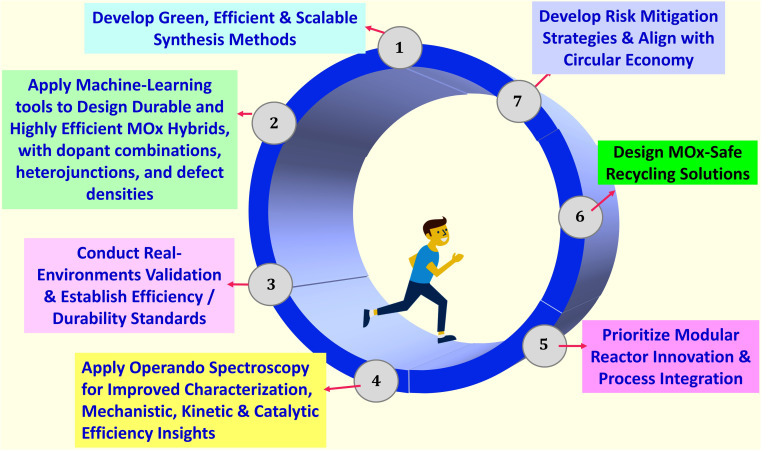
Summary of prospectives of the synthesis, characterization and applications of MOx in photocatalytic processes.

By addressing these advanced research directions, we can push the boundaries of MOx-based nanomaterials and pave the way for their widespread implementation in addressing critical environmental and energy challenges. A key emphasis should be placed on bridging the gap between fundamental research and practical applications. Fig. 15 shows a summary of perspectives of the synthesis, characterization and applications of MOx in photocatalytic processes.

## Data availability

The data analyzed in this review article are from previously published studies. The specific datasets and sources are cited throughout the manuscript and listed in the reference section. Readers can access the underlying data from the original published sources as cited. The authors confirm that they did not have any special access privileges to these datasets.

## Conflicts of interest

There are no conflicts to declare.
